# Targeting Heat Shock Protein 27 and Fatty Acid Oxidation Augments Cisplatin Treatment in Cisplatin-Resistant Ovarian Cancer Cell Lines

**DOI:** 10.3390/ijms241612638

**Published:** 2023-08-10

**Authors:** James Patrick Heiserman, Zenab Minhas, Elahe Nikpayam, Dong-Joo Cheon

**Affiliations:** Department of Regenerative and Cancer Cell Biology, Albany Medical College, Albany, NY 12208, USA; jimheiserman@gmail.com (J.P.H.); nikpaye@amc.edu (E.N.)

**Keywords:** ovarian cancer, cisplatin resistance, HSP27, FAO, ROS

## Abstract

Most ovarian cancer patients develop recurrent cancers which are often resistant to commonly employed chemotherapy agents, such as cisplatin. We have previously shown that the inhibition of heat shock protein 27 (HSP27) or fatty acid oxidation (FAO) sensitizes cisplatin-resistant ovarian cancer cell lines to cisplatin and dual inhibition of both HSP27 and FAO induces substantial cell death in vitro. However, it is unclear how HSP27 and FAO promote cisplatin resistance, and if dual inhibition of both HSP27 and FAO would augment cisplatin treatment in vivo. Here we showed that HSP27 knockdown in two cisplatin-resistant ovarian cancer cell lines (A2780CIS and PEO4) resulted in more ROS production upon cisplatin treatment. HSP27-knockdown cancer cells exhibited decreased levels of reduced glutathione (GSH) and glucose6phosphate dehydrogenase (G6PD), a crucial pentose phosphate pathway enzyme. ROS depletion with the compound N-acetyl cysteine (NAC) attenuated cisplatin-induced upregulation of HSP27, FAO, and markers of apoptosis and ferroptosis in cisplatin-resistant ovarian cancer cell lines. Finally, inhibition of HSP27 and FAO with ivermectin and perhexiline enhanced the cytotoxic effect of cisplatin in A2780CIS xenograft tumors in vivo. Our results suggest that two different cisplatin-resistant ovarian cancer cell lines upregulate HSP27 and FAO to deplete cisplatin-induced ROS to attenuate cisplatin’s cytotoxic effect.

## 1. Introduction

Ovarian cancer is the fifth leading cause of cancer mortalities for women in the United States [1]. Cisplatin is an FDA-approved platinum-based chemotherapy drug for the treatment of ovarian cancer [2]. Although 85% of ovarian cancer patients initially respond to cisplatin treatment, unfortunately 75% of patients will subsequently develop recurrent ovarian cancer, which likely will no longer respond to subsequential cisplatin treatment [3]. Considering this, it is important to understand the mechanisms of cisplatin resistance to develop a novel therapy to overcome cisplatin resistance.

Previously, our group reported that COL11A1, a gene encoding collagen α1(XI), is highly upregulated in cisplatin-resistant ovarian cancers [4,5]. We further showed that collagen α1(XI) promotes cisplatin resistance by upregulating heat shock protein 27 (HSP27) or fatty acid oxidation (FAO) in ovarian cancer cells [4,5]. Interestingly, the genetic and pharmaceutical inhibition of HSP27 upregulated carnitine palmitoyltransferase I alpha (CPT1A), the rate-limiting enzyme of FAO, to sustain ovarian cancer cell survival during cisplatin treatment [5]. Dual inhibition of HSP27 and FAO, with ivermectin (an inhibitor of phosphorylated HSP27 [6]) and perhexiline (a CPT protein inhibitor [7]), induced substantial apoptosis in cisplatin-resistant ovarian cancer cells in vitro [5]. However, it is still unclear how HPS27 and FAO confer cisplatin resistance, as well as if their dual inhibition in vivo would enhance cisplatin treatment.

HSP27 is a 27-kDa protein chaperone that has been linked to drug resistance across many cancer types [8,9,10,11]. HSP27 is considered a pro-survival signaling molecule by activating Akt and NF-kB signaling [12,13]. However, it is still unclear how HSP27 confers cisplatin resistance. Several studies have demonstrated that HSP27 functions as an antioxidant, thereby decreasing the levels of cellular reactive oxygen species (ROS) [14,15,16,17]. Of note, cisplatin has been shown to induce cancer cell apoptosis by inducing cytotoxic levels of ROS [18,19]. Excessive ROS can also induce ferroptosis, an iron-dependent cell death mediated by ROS and lipid peroxidation. Glutathione peroxidase 4 (GPX4) is an enzyme that detoxifies cytotoxic lipid peroxides into lipid alcohols [20], and a loss of GPX4 expression serves as a marker of ferroptosis. It has been shown that cisplatin can also induce ferroptosis [21] and HSP27 attenuates ferroptosis [22]. HSP27 functions as a negative regulator of ROS through two known mechanisms: first by inhibition of transferring an iron transporter [15], and second by binding directly to and enhancing glucose-6-phosphate dehydrogenase (G6PD) activity [16,17]. G6PD is a crucial enzyme in the pentose phosphate pathway (PPP), the main source of nicotinamide adenine dinucleotide phosphate (NADPH). NADPH is then used to reduce oxidized glutathione (GSSG) to reduced glutathione (GSH) [23], a major antioxidant that detoxifies cytotoxic levels of ROS [24].

FAO is a metabolic process by which fatty acids are catabolized to produce biomolecules such as ATP, deoxyribonucleotide triphosphates (dNTPs), NADH, and NADPH [25]. Fatty acids are transported into a cell through cell surface proteins such as fatty acid translocase (CD36) [26]. Fatty acids are then further modified and enter the mitochondria through carnitine palmitoyltransferase-1 (CPT1), the rate-limiting enzyme of FAO [27]. After transport into the mitochondria, fatty acids are processed through β-oxidation to produce biomolecules such as acetyl-CoA, which can then be processed in the TCA cycle to produce NADH and NADPH [28,29].

In this study, we demonstrate that cisplatin-resistant ovarian cancer cells upregulate HSP27 and FAO to attenuate cisplatin-induced ROS and cell death. HSP27-knockdown cancer cells exhibit higher levels of mitochondrial and cellular ROS, as well as decreased levels of GSH and G6PD. We also found that ROS depletion attenuates cisplatin-induced HSP27, FAO, apoptosis, and ferroptosis in cisplatin-resistant ovarian cancer cells. Finally, we showed that dual inhibition of HSP27 and FAO increased the efficacy of cisplatin treatment in A2780CIS xenograft tumors.

## 2. Results

### 2.1. HSP27 Inhibits Cisplatin-Induced ROS and Ferroptosis in Cisplatin-Resistant Ovarian Cancer Cells

**Figure 1:**
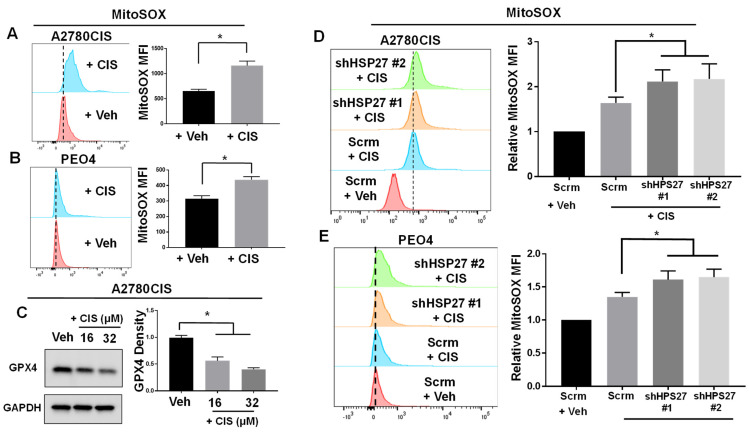
HSP27 knockdown increases cisplatin-induced ROS in cisplatin-resistant ovarian cancer cells. (**A**,**B**) Flow cytometry of A2780CIS and PEO4 cells treated with vehicle (Veh) or 16 μM cisplatin (CIS) and then stained with MitoSOX dye. Quantification of MitoSOX Mean Fluorescence Intensity (MFI) was shown to right. (**C**) Western blot of GPX4 (normalized to GAPDH) in A2780CIS cells treated with vehicle (Veh) or cisplatin (16 or 32 μM). (**D**,**E**) Flow cytometry of A2780CIS (**D**; N = 4) or PEO4 (**E**) cells. Scrambled control or HSP27 knockdown cells were treated with vehicle (Veh) or 16 μM CIS and stained with MitoSOX. Quantification of relative MitoSOX MFI on graphs to right. Unpaired two-tailed two-sample *t*-test (for **A**,**B**) or one-way ANOVA (For **C**–**E**). Data are presented as mean ± SD. N = 3 unless otherwise indicated. *, *p* < 0.05. Uncropped Western blot images are displayed in Figure S12.

Previous reports have demonstrated that cisplatin induces mitochondrial and cellular ROS [18,19]. To confirm these results, we assessed the levels of mitochondrial and cellular ROS in two cisplatin-resistant ovarian cancer cell lines. We treated A2780CIS and PEO4 cell lines, which are cisplatin-resistant ovarian cancer cell lines [30,31], with cisplatin, and measured mitochondrial and cellular ROS with MitoSOX and CellROX fluorescent dyes, respectively. We found that cisplatin induces mitochondrial and cellular ROS in both cell lines (Figure 1A,B and Figure S1A,B), confirming the results of previous studies. Since cisplatin is reported to induce ferroptosis [21], we also checked whether cisplatin induces ferroptosis in A2780CIS cells. We observed decreased levels of GPX4 (indicative of ferroptosis [32]) in cisplatin-treated A2780CIS cells (Figure 1C), again confirming previous reports that cisplatin induces ferroptosis. This data demonstrates that cisplatin induces ROS and ferroptosis in cisplatin-resistant ovarian cancer cell lines.

In a previous study, we showed that HSP27 expression was induced by cisplatin treatment in A2780CIS cells [5]. Furthermore, we also observed that HSP27 knockdown sensitized A2780CIS cells to cisplatin treatment [5]. Additionally, we found that A2780CIS cells exhibited higher expressions of HSP27 compared to their cisplatin-sensitive parental cell line, A2780 cells [5]. In line with this, we also examined HSP27 expression levels in another cisplatin-resistant ovarian cell line, PEO4 cells, compared to its cisplatin-sensitive counterpart, PEO1 cells. We found that HSP27 expression was increased in PEO4 cells relative to cisplatin-sensitive PEO1 cells (Figure S2A). Importantly, we also found that HSP27 conferred cisplatin resistance to A2780CIS cells, and not A2780 cells, when these cells were assessed for cleaved capase-3 and general cell viability when treated with 16 μM cisplatin (Figure S2B,C). HSP27 has been shown to inhibit ROS generation by activating the pentose phosphate pathway (the main pathway that produces cellular NADPH) through a direct binding to G6PD [16,17]. Since cisplatin induces ROS and HSP27 inhibits ROS generation, we tested whether HSP27-knockdown ovarian cancer cells exhibit higher levels of ROS when treated with cisplatin. For this, we stably knocked down HSP27 in A2780CIS and PEO4 cells (the HSP27 knockdown efficiency of ovarian cancer cell lines tested was confirmed in Figures S3A–C and S13A–C), treated them with cisplatin, and measured mitochondrial and cellular ROS levels with MitoSOX and CellROX dyes, respectively. We found that cisplatin-treated HSP27 knockdown A2780CIS and PEO4 cells had increased levels of mitochondrial ROS, and the A2780CIS cells also exhibited higher levels of cellular ROS compared to their scramble control counterparts (Figure 1D,E and Figure S1B). Of note, we did not see a difference in mitochondrial or cellular ROS between HSP27- knockdown A2780CIS and PEO4 cells and their scrambled controls without cisplatin treatment (Figure S4A–C).

Collectively, these results demonstrate that cisplatin induces ROS and ferroptosis, and HSP27 knockdown further increases cisplatin-induced ROS in A2780CIS and PEO4 cisplatin-resistant ovarian cancer cells.

### 2.2. ROS Depletion Attenuates HSP27 Induction and Cytotoxic Effects of Cisplatin

**Figure 2:**
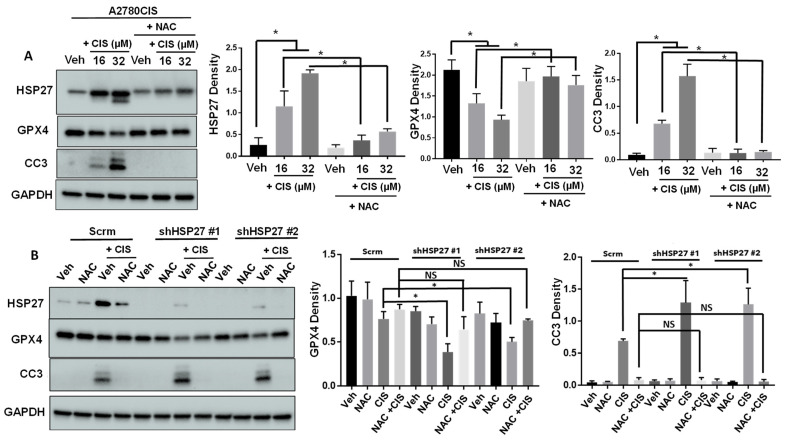
ROS depletion by NAC attenuates cisplatin-induced HSP27 expression and cell death (**A**,**B**) Western blot of total HSP27, GPX4, or cleaved caspase-3 (CC3) in A2780CIS (**A**) or scrambled control (scrm) and HSP27-knockdown A2780CIS cells (**B**) treated with DMSO (Veh), 16 µM cisplatin (CIS), 2.5 mM NAC, or combination (CIS + NAC). Quantification of band intensities (normalized to GAPDH) on the right. One-way ANOVA. Mean ± SD. *, *p* value < 0.05. N = 3. NS, not significant. Uncropped Western blot images are displayed in Figure S14.

We next sought to determine whether ROS mediates the cytotoxic effects of cisplatin. To do this, we employed the compound N-Acetyl-Cysteine (NAC) which replenishes levels of reduced glutathione (GSH), thereby depleting ROS [33]. NAC attenuated cisplatin-induced cell death (indicated by a loss of cell viability) in A2780CIS and PEO4 cells (Figure S5A,B). We also observed that NAC attenuated cisplatin-induced apoptosis and ferroptosis, marked by decreased levels of cleaved caspase-3 and rescuing of GPX4 expression, respectively (Figure 2A and Figure S6A). This data reinforces the hypothesis that ROS, at least partially, mediates the cytotoxic effects of cisplatin. Interestingly, NAC treatment also attenuated cisplatin-induced HSP27 upregulation in A2780CIS cells (Figure 2A). However, in PEO4 cells, NAC attenuated cisplatin-induced upregulation of phosphorylated HSP27 (at Serine 82), but not total levels of HSP27 (Figure S6A). This data suggests that cisplatin induction of HSP27 may be mediated by ROS.

Next, we tested whether NAC treatment could rescue a loss of viable HSP27 knockdown cancer cells upon cisplatin treatment. HSP27-knockdown A2780CIS and PEO4 cells exhibited increased levels of apoptosis and ferroptosis compared to control cells when treated with cisplatin alone (Figure 2B and Figure S6B). However, when cells were treated with both cisplatin and NAC, there was no difference in the levels of apoptosis and ferroptosis between the control and HSP27-knockdown cells (Figure 2B and Figure S6B). These results demonstrate that the effects of the loss of HSP27 can be rescued by ROS depletion in a cisplatin-treated context.

### 2.3. HSP27-Knockdown Ovarian Cancer Cells Exhibit Decreased Levels of GSH and G6PD

**Figure 3:**
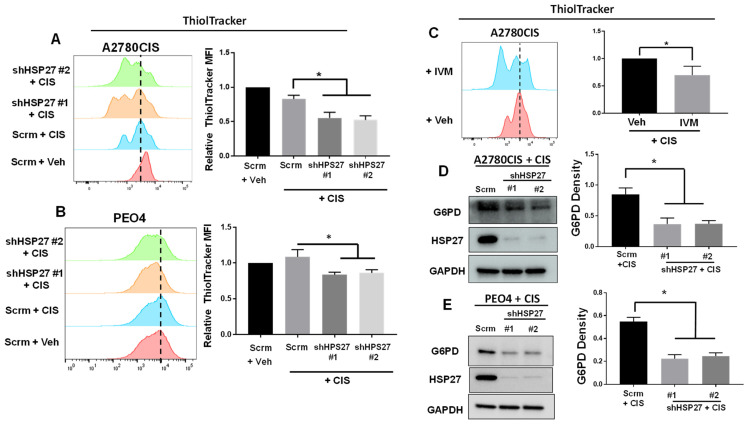
HSP27-knockdown cancer cells exhibit decreased levels of reduced thiols and G6PD. (**A**,**B**) Flow cytometry of scramble control (Scrm) or HSP27-knockdown A2780CIS (**A**) and PEO4 (**B**) cells treated with vehicle (Veh) or 16 μM cisplatin (CIS) and stained with ThiolTracker. Quantification of relative ThiolTracker MFI on the right. (**C**) Flow cytometry of A2780CIS cells treated with vehicle (Veh) + CIS or 1.5 μM ivermectin (IVM) + CIS and assessed with ThiolTracker. Quantification of ThiolTracker MFI on the right. (**D**,**E**) Western blot of G6PD in A2780CIS-scrm or A2780CIS-shHSP27 cells treated with 16 µM CIS. Quantification of G6PD (normalized to GAPDH) on the right. One-way ANOVA. Mean ± SD. *, *p* value < 0.05. N = 3. Uncropped Western blot images are displayed in Figure S15.

HSP27 provides cellular protection against ROS through two major mechanisms, one of which is the activation of the pentose phosphate pathway through direct binding to G6PD [16,17]. The pentose phosphate pathway is a major source of NADPH, which is a crucial cofactor for the reduction of glutathione from its oxidized (GSSG) to reduced form (GSH) [23]. Considering this, we sought to determine whether HSP27-knockdown ovarian cancer cells decrease levels of GSH and G6PD when treated with cisplatin. To measure GSH, we treated HSP27-knockdown and control A2780CIS and PEO4 cells with cisplatin, and stained them with ThiolTracker, a fluorescent dye that stains reduced thiols (primarily GSH) [24]. In agreement with the literature, HSP27-knockdown A2780CIS and PEO4 cells exhibited decreased levels of GSH compared to control cells with or without cisplatin treatment (Figure 3A,B and Figure S7A,C). Similarly, A2780CIS cells treated with ivermectin (an HSP27 inhibitor [6]) also exhibited decreased levels of GSH with or without cisplatin treatment (Figure 3C and Figure S7B). These results demonstrate that HSP27 facilitates the production of GSH (in both basal condition and cisplatin-treated condition) in cisplatin-resistant ovarian cancer cells.

Since HSP27 has been shown to bind to and enhance G6PD activity [16,17], we next assessed the expression of G6PD in HSP27-knockdown A2780CIS and PEO4 cells. We found that G6PD expression was reduced in HSP27-knockdown cells relative to control cells (Figure 3D,E). These results suggest that the pentose phosphate pathway is compromised in HSP27-knockdown ovarian cancer cells, explaining why GSH levels could be decreased in HSP27-knockdown cells.

### 2.4. HSP27-Knockdown Ovarian Cancer Cells Exhibit Increased Levels of Lipid Peroxidation, and NAC Attenuates Cisplatin-Induced FAO Marker Upregulation

We have shown that in response to cisplatin treatment, HSP27-knockdown ovarian cancer cells upregulate CPT1A [5], the rate-limiting enzyme of FAO [34]. We also found that dual inhibition of HSP27 and CPT proteins, with the drugs ivermectin and perhexiline (a CPT inhibitor [7]), respectively, drastically induced apoptosis in cisplatin-resistant ovarian cancer cells [5]. Thus, it was not surprising that cisplatin-treated HSP27 knockdown ovarian cancer cells exhibited higher levels of TMRE fluorescence, which is indicative of increased electron transport chain/FAO activity [35] (Figure S8A). Similar results were obtained with ivermectin-treated ovarian cancer cells (Figure S8A). Of note, the total mitochondrial content (measured by MitoTracker fluorescent dye) did not change between these groups (Figure S8B). Additionally, there was no difference in TMRE nor MitoTracker fluorescent intensities between HSP27-inhibited cells and control cells when they were not treated with cisplatin (Figure S8C,D).

**Figure 4:**
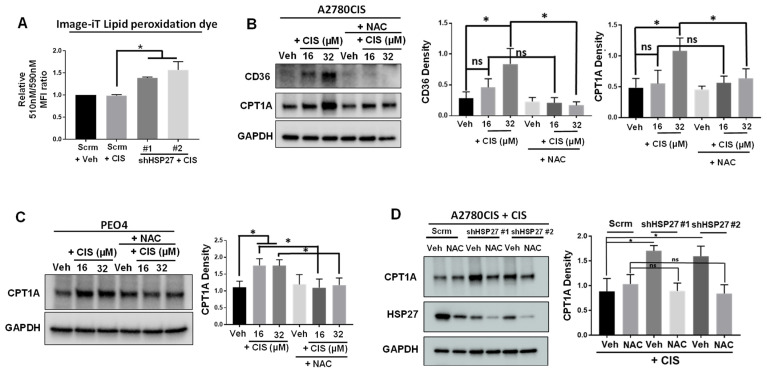
HSP27 knockdown increases lipid peroxidation in cisplatin-treated cells, and NAC attenuates cisplatin induction of FAO markers. (**A**) Flow cytometry of scramble control or HSP27 knockdown A2780CIS cells treated with vehicle (Veh) or 16 μM Cisplatin (CIS) and then assessed with Image-iT Lipid peroxidation dye. Graph shows quantification of 510 nM to 590 nM ratio emitted from lipid peroxidation dye. (**B**,**C**) Western blot of CD36 in A2780CIS (**B**) cells and CPT1A in A2780CIS (**B**) and PEO4 (**C**) cells treated with DMSO (Veh), 16 µM or 32 µM cisplatin (CIS), 2.5 mM NAC, or combination (CIS + NAC). Quantification of CD36 and CPT1A (normalized to GAPDH) on the right. (**D**) Western blot of CPT1A and HSP27 in scramble control and HSP27-knockdown A2780CIS cells treated with 16 µM CIS or CIS + NAC. Quantification of CPT1A (normalized to GAPDH) on the right. One-way ANOVA. Mean ± SD. *, *p* value < 0.05. NS, not significant. Uncropped Western blot images are displayed in Figure S16.

Next, we sought to determine if ROS attenuation through NAC treatment could attenuate cisplatin-induced FAO upregulation in cisplatin-treated ovarian cancer cells. For this, we measured the expression of the FAO markers CPT1A and CD36 in cisplatin-treated A2780CIS and PEO4 cells, with or without NAC treatment. We observed that induction of CD36 and CPT1A was only achieved at a higher concentration of cisplatin (32 μM) in A2780CIS cells, which was attenuated by NAC treatment (Figure 4B). In PEO4 cells, cisplatin-induced CPT1A expression at both 16 and 32 μM, which was attenuated by NAC treatment (Figure 4C). Since we previously observed an induction of CPT1A in HSP27-knockdown A2780CIS cells [5], we also assessed the levels of CPT1A in cisplatin-treated HSP27-knockdown A2780CIS cells with or without NAC treatment. We found that cisplatin-induced CPT1A expression was attenuated by NAC treatment in HSP27 knockdown A2780CIS cells (Figure 4D).

Since cisplatin-treated HSP27-knockdown ovarian cancer cells exhibit increased levels of ferroptosis than control cells (Figure 2B), we measured the levels of lipid peroxidation (another marker of ferroptosis [36]) in these cells. For this, we employed Image-iT Lipid Peroxidation dye, a fluorescent dye that measures lipid peroxidation through the measurement of a dye emission shift from 590 nM to 510 nM, which indicates lipid peroxidation. We found that cisplatin-treated HSP27-knockdown cancer cells exhibited significantly increased levels of lipid peroxidation, as indicated by a significantly higher ratio of a 510 nM to 590 nM emission from the Image-iT Lipid Peroxidation dye (Figure 4A and Figure S9A). Importantly, there was no difference in the levels of 510 nM and 590 nM fluorescence from this dye between HSP27-knockdown cells and control cells when they were not treated with cisplatin (Figure S9B,C). These data suggest that HSP27-knockdown ovarian cancer cells exhibit higher levels of lipid peroxidation. Collectively, these results suggest that cisplatin may induce FAO through an ROS-based mechanism in cisplatin-resistant ovarian cancer cells. FAO upregulation might be another way for cisplatin-resistant ovarian cancer cells to generate NADPH to manage and deplete high levels of ROS.

### 2.5. Dual Inhibition of HSP27 and FAO Augments the Antitumor Effect of Cisplatin In Vivo

Our previous in vitro data showed that dual inhibition of HSP27 and FAO with ivermectin and perhexiline resulted in the substantial cell death of A2780CIS cells [5]. In line with our previous results, we also observed that the combination of ivermectin and perhexiline resulted in significant apoptosis of PEO4 cells compared to vehicle control or monotherapy (Figure S10A,B). To test the efficacy of this drug combination in vivo, we injected 5 million A2780CIS cells subcutaneously into both rear flanks of NCr female nude mice. After 12 to 18 days, palpable tumors are formed, ranging from 0.3 cm^3^ to 0.5 cm^3^ in size. Then, mice were evenly distributed across five treatment groups to ensure no difference in starting tumor size across treatment groups (Figure S11A).

**Figure 5:**
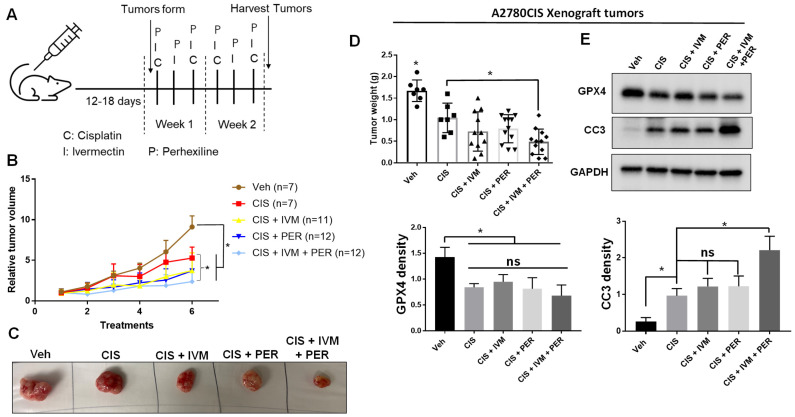
Dual inhibition of HSP27 and FAO augments the antitumor effect of cisplatin in vivo. (**A**) A schematic depiction of A2780CIS xenograft experiment. Once subcutaneous tumors are formed 12–18 days after tumor cell injection, mice were distributed to five groups and treated with: vehicle (Veh), 3 mg/mL cisplatin (CIS), 3 mg/mL cisplatin and 3 mg/mL ivermectin (CIS + IVM), 3 mg/mL cisplatin and 8 mg/mL perhexiline (CIS + PER), or all three drugs (CIS + IVM + PER). (**B**) Relative tumor volume (measured in cm^3^, normalized to starting tumor volume at day 1 of the treatment) over the course of treatment. N = number of tumors. One-way ANOVA. (**C**) Representative images of A2780CIS xenograft tumors from each treatment group. (**D**) Wet weights of excised subcutaneous tumors (in grams (g)) after 6 treatment cycles. Each dot indicates an individual tumor. (**E**) Western blot of GPX4 and cleaved caspase-3 (CC3) in A2780CIS xenograft tumors. Quantification of markers normalized to GAPDH is shown below. One-way ANOVA. Mean ± SD. *, *p* value < 0.05. ns, not significant. Uncropped Western blot images are displayed in Figure S17.

After distribution into five groups, mice then received the following treatments in each group; (1) vehicle (mice were treated with the same volume of saline/DMSO and frequency as other treatments), (2) cisplatin alone (3 mg/kg; twice a week), (3) cisplatin with ivermectin (3 mg/kg; three times a week), (4) cisplatin with perhexiline (8 mg/kg; three times a week), and (5) cisplatin, ivermectin, and perhexiline treatments (Figure 5A). Cisplatin was included in all treatment groups because our in vitro data showed that cisplatin-induced ROS exacerbated in HSP27-knockdown A2780CIS cells (Figure 1 and Figure 2). After six treatments, vehicle tumors were significantly larger compared to all other treatment groups (Figure 5B–D). However, within the cisplatin-treated groups, only the triple therapy group (CIS + IVM + PER) exhibited significantly smaller tumors than the cisplatin (CIS) group (Figure 5B–D). Additionally, only the triple therapy group exhibited more apoptotic tumors compared to the cisplatin group (Figure 5E). All cisplatin-treated tumors exhibited increased ferroptosis (indicated by decreased GPX4 levels) compared to the vehicle-treated tumors (Figure 5E), although there was no difference in GPX4 expression across the cisplatin-treated groups. Importantly, there was no change in mouse body weight during the six treatments of these drugs (Figure S11B), suggesting that mice could tolerate the treatments. This data demonstrates that dual inhibition of HSP27 and FAO with ivermectin and perhexiline may be a viable strategy to treat cisplatin-resistant ovarian tumors.

### 2.6. Differential Expression of HSP27 and CPT1A in A2780CIS Xenograft Tumors

**Figure 6:**
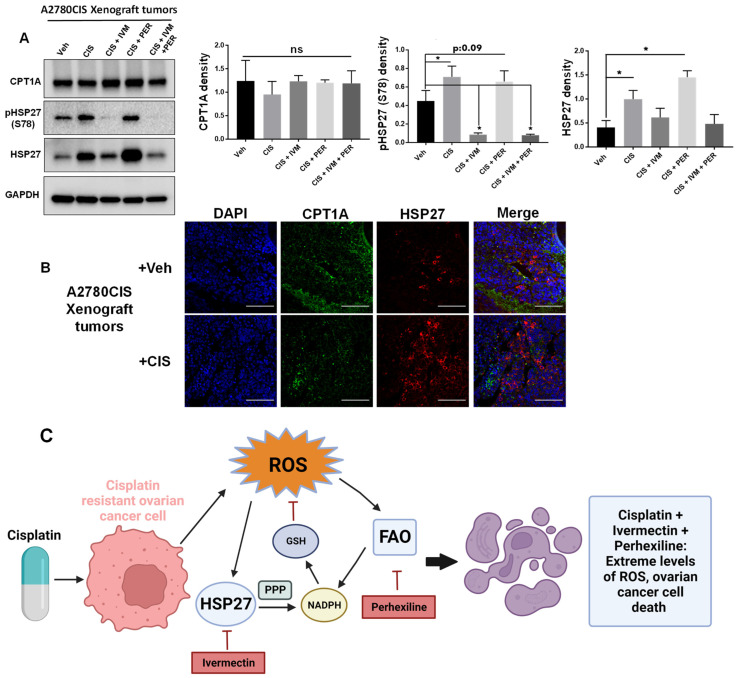
Differential expression of HSP27 and CPT1A in A2780CIS xenograft tumors. (**A**) Western blot of CTP1A, pHSP27 (S78), and total HSP27 in A2780CIS xenograft tumors. Quantification of markers normalized to GAPDH on the right. One-way ANOVA. Mean ± SD. *, *p* value < 0.05. ns, not significant (**B**) Representative immunofluorescence images of DAPI, CPT1A, and HSP27 in vehicle or cisplatin-treated A2780CIS xenograft tumors. Scale bars = 100 μM (**C**) Proposed model of how dual inhibition of HSP27 and FAO promotes the antitumor effect of cisplatin. Images created with Biorender. Uncropped Western blot images are displayed in Figure S17.

To gain further insight into the effects of the triple therapy in vivo, we measured the levels of CPT1A and HSP27 (phosphorylated and total) in xenograft tumors from the five treatment groups using Western blot. We found that there was no difference in CPT1A expression across treatment groups (Figure 6A). However, the levels of phosphorylated HSP27 (at serine 78, which is inhibited by ivermectin [6]) were significantly decreased in tumors treated with ivermectin (CIS + IVM, CIS + IVM + PER) compared to vehicle control tumors (Figure 6A). We also found that the levels of total HSP27 were significantly upregulated in tumors treated with cisplatin (CIS) or combination therapy with cisplatin and perhexiline (CIS + PER) compared to vehicle control tumors (Figure 6A). This data suggests that cisplatin increases phosphorylated and total HSP27 expression while ivermectin attenuates phosphorylated and total HSP27 expression in vivo.

We also examined the expression of CPT1A and HSP27 in A2780CIS xenograft tumors using immunofluorescence. We found that the expression of CPT1A and HSP27 was partially exclusive from each other in A2780CIS xenograft tumors (Figure 6B). CPT1A staining was uniform throughout the tumor sections with low to moderate intensity. In contrast, HSP27 staining was intense in some clusters of cancer cells but typically HSP27 staining showed low to moderate intensity in other areas (Figure 6B). These results suggest that tumor cells that express high levels of either HSP27 or FAO may be exclusive from one another. This observation provides a possible explanation as to why combination therapy of ivermectin and perhexiline is effective in vitro and in vivo as these drugs might target two different cancer cell populations. Regardless, our results demonstrate that dual inhibition of HSP27 and FAO promotes the antitumor effect of cisplatin, potentially through increasing ROS in cancer cells (Figure 6C). Our study also suggests a combination therapy of ivermectin and perhexiline as a viable strategy to treat cisplatin-resistant ovarian cancer.

## 3. Discussion

HSP27 is a chaperone molecule that has been associated with therapy resistance across multiple cancer types [8,9,10,11]. In this study, we showed that HSP27 attenuates cisplatin-induced cell death by decreasing cellular and mitochondrial ROS levels through activation of the pentose phosphate pathway and GSH generation. We also showed that cisplatin induces the expression of HSP27 and FAO markers (i.e., CPT1A, CD36) in cisplatin-resistant ovarian cancer cells through ROS-dependent mechanisms as NAC treatment attenuates cisplatin induction of HSP27 and FAO. Finally, we demonstrated that dual inhibition of HSP27 and FAO with ivermectin and perhexiline enhanced the antitumor effect of cisplatin in vivo.

First, we must address why HSP27 inhibition needs cisplatin treatment to show phenotypic differences (e.g., increased mitochondrial potential, increased mitochondrial and cellular ROS levels) in cisplatin-resistant ovarian cancer cells. One possibility is that HSP27 might need to be induced to a certain level of expression by cisplatin treatment to exhibit obvious cellular phenotypes. Alternatively, since HSP27 is a regulator of ROS [14,15,16,17] and cisplatin induces ROS [18,19], the loss of HSP27 in a cisplatin-treated context might cause more dramatic phenotypes than control conditions due to potentially cytotoxic levels of ROS.

Our previous [5] and present study has also demonstrated that FAO may be a critical compensatory mechanism induced by HSP27 inhibition to maintain cell survival of cisplatin-resistant ovarian cancer cells. This is consistent with a previous study showing that HSP90-inhibited prostate cancer cells induce FAO to maintain cell survival [37]. Other studies showed that ivermectin-treated activate AMPK in hepatocytes and upregulate CPT1A in preadipocytes [38,39]. Conversely, CPT2 knockdown induced HSP27 expression in hepatocytes [40], suggesting that HSP27 and FAO compensate for each other when one is inhibited. FAO breaks down long chain fatty acids into molecules of acetyl-CoA in the mitochondria, which is oxidized through the TCA cycle and the electron transport chain to produce ATP. In addition to ATP production, FAO is also involved in the generation of cytosolic NADPH, an important co-factor for generating GSH. Thus, FAO may compensate for the loss of HSP27 (or vice versa) through generation of NADPH to support redox homeostasis in cisplatin-resistant ovarian cancer cells. Additional studies are needed to determine what cellular factors FAO would supply to deplete cisplatin-induced ROS in the absence of HSP27.

It remains to be determined how FAO is upregulated in response to HSP27 inhibition post cisplatin treatment. Our data from this current study and our previous work [5] showed that cisplatin-treated HSP27-knockdown cancer cells exhibit higher levels of ROS and AMPK. There is evidence showing that ROS can activate AMPK [41], a master kinase that can activate FAO [34]. Thus, we speculate that the combination of HSP27 inhibition and cisplatin treatment might boost ROS generation and subsequent AMPK activation, thereby upregulating FAO. Of note, AMPK is also known to inhibit heat shock factor-1 (HSF1), a transcription factor that upregulates HSP27 expression [42]. Another possibility is that the combination of cisplatin and HSP27 inhibition might select cancer cell populations that exhibit high levels of FAO. Indeed, our in vivo immunofluorescence data show that HSP27 and FAO expression is somewhat exclusive to each other in A2780CIS xenograft tumors. Also, many cancer cell lines maintain a heterogenous population of cancer cells [43]. Thus, it is possible that targeting HSP27-high cancer cell populations might selectively enrich FAO-high cancer cell populations. Future studies are warranted to determine how HSP27 inhibition leads to FAO upregulation in cisplatin-resistant ovarian cancer cells.

It is also interesting why HSP27-knockdown ovarian cancer cells exhibit higher levels of CPT1A, the rate limiting enzyme of FAO, as well as higher lipid peroxidation upon cisplatin treatment. Other reports have shown that monounsaturated fatty acids (MUFAs) protect against ferroptosis, whereas polyunsaturated fatty acids (PUFAs) may promote ferroptosis depending on context [44,45]. It is tempting to speculate that the HSP27 knockdown ovarian cancer cells consume specific lipid species, such as MUFAs, to generate NADPH in order to attenuate increased levels of ROS and lipid peroxidation. Future studies are warranted to study the role of specific lipid species in ovarian cancer cisplatin resistance.

We also need to address the differences between our in vitro and in vivo data. Our in vitro data showed that cisplatin-treated HSP27-knockdown A2780CIS cells induce CPT1A expression compared to scrambled control. However, in A2780CIS xenograft tumors, cisplatin and ivermectin treatment did not induce CPT1A expression. This could be due to the fact that in vitro experiments were performed on a shorter timescale (72 h) as opposed to the two-week treatment period in vivo, where the tumor cells may have adapted to cisplatin and ivermectin treatments. Alternatively, we might need to increase the concentration or treatment frequency of ivermectin to induce CPT1A expression in tumors. Since FAO is required for the in vivo growth of breast cancer cells [46], it is also possible that A2780CIS cells might require CPT1A expression for cell survival in vivo. Regardless, our results showed that dual inhibition of HSP27 and FAO enhances the antitumor effect of cisplatin in vitro and in vivo.

In conclusion, our study demonstrates that cisplatin-resistant ovarian cancer cell lines upregulate HSP27 and FAO to deplete cisplatin-induced ROS to attenuate the cytotoxic effect of cisplatin. Dual inhibition of HSP27 and FAO with ivermectin and perhexiline enhances the cytotoxic effect of cisplatin, serving as a promising therapeutic strategy for cisplatin-resistant ovarian cancer. Future studies warrant the evaluation of dual inhibition of HSP27 and FAO in other cisplatin-resistant cell lines and patient samples.

## 4. Materials and Methods

### 4.1. Cell Culture

PEO1, PEO4, and A2780CIS cell lines were purchased from SIGMA (St. Louis, MO, USA). Lenti-X 293T cell line was purchased from Takara Bio (Kusatsu, Shiga, Japan). A2780CIS cells were cultured in RPMI-1640 medium supplemented with 10% FBS and 100 units/mL of penicillin and 100 µg/mL streptomycin. PEO1 and PEO4 cells were cultured in RPMI-1640 medium supplemented with 2 mM glutamine, 2 mM sodium pyruvate, and the same concentrations of FBS and penicillin/streptomycin as described above. All cells were maintained in an incubator at 37 °C with 5% CO_2_. All cell lines were authenticated before experimentation and tested negative for mycoplasma.

For experiments with N-Acetyl-L-cysteine (NAC; Sigma, St. Louis, MO, USA), cells were pre-treated with 2.5 mM NAC thirty minutes prior to saline or cisplatin treatment.

### 4.2. Cell Viability Assays

MTT (3-(4,5-dimethylthiazol-2-yl)-2,5-diphenyl-2H-tetrazolium bromide, Millipore Sigma; #11465007001, St. Louis, MO, USA) and acid phosphatase (Millipore Sigma; #4876, St. Louis, MO, USA) assays were used to assess cell viability using a Glomax Explorer Microplate Reader (Promega; Madison, WI, USA) following manufacturer’s instructions.

### 4.3. Lentiviral shRNA Knockdown

Stable gene knockdown was performed using shRNAs directed against HSPB1 (the gene name for HSP27) as described previously [4,5]. All reagents and TRCN numbers for shRNAs are listed in a previous publication [5]. Lipofectamine 2000 (Life technologies; #11668019, Carlsbad, CA, USA) was used as a liposome carrier to co-transfect of pCMV-ΔR 8.2 (Addgene; #8455, Watertown, MA, USA), pCMV-VSVG (Addgene; #8454, Watertown, MA, USA), and a lentiviral construct containing shRNA for HSPB1 (Sigma-Aldrich; St. Louis, MO, USA) into Lenti-X 293T cells [5]. Lentivirus-containing media was collected 48 h later and filtered through a 0.45 µm PVDF low protein-binding membrane filter (Celltreat, Pepperell, MA, USA). A2780CIS and PEO4 cells were incubated in the lentivirus-containing medium supplemented with 8 µg/mL polybrene (EMD Millipore; #TR-1003-G, Burlington, MA, USA) for 72 h at 37 °C with 5% CO_2_. HSP27-knockdown cells (or scramble control cells) were then selected with 5 µg/mL puromycin (ThermoFisher; #A1113803, Waltham, MA, USA). Knockdown efficiency is shown in Figure S3A–C.

### 4.4. Immunoblotting

Western blotting was performed to analyze protein expression in cell and tumor lysates as described previously [4,5]. Briefly, cells were collected through trypsinization, washed with PBS, and lysed using RIPA cell lysis buffer (Sigma; #R0278, St. Louis, MO, USA) supplemented with complete mini protease inhibitor cocktail (Sigma; #11836170001, St. Louis, MO, USA) and PhosSTOP (Sigma; #4906845001, St. Louis, MO, USA). For tumor samples, tumors were snap frozen with liquid nitrogen and stored at −80 °C. Then, small pieces (approximately 0.1–0.2 g in weight) of tumor samples were homogenized with the PowerGen 125 homogenizer (Fisher Scientific, Hampton, NH, USA) in RIPA buffer containing 1 mM phenylmethanesulfonyl fluoride (PMSF; Sigma; #P7626-5G, St. Louis, MO, USA) and phosphatase inhibitor cocktail II (Abcam; ab201113, Cambridge, UK). Protein concentrations from cell and tumor lysates were determined with Pierce’s BCA Protein Assay Kit (ThermoFisher; #23225, Waltham, MA, USA). Samples containing approximately 10 to 25 µg of protein were run on a gradient (4–20%) Mini-PROTEAN^®^ TGX Stain-Free™ Protein Gel (Bio-Rad; #4568094, Hercules, CA, USA). Samples were then transferred onto PVDF membranes followed by blocking for 1 h (with PBST + 5% BSA). Blots were then incubated in primary antibodies overnight, washed three times with PBST, and then incubated in secondary antibody for 1 h (employed antibodies are listed in Table S1). Clarity western ECL reagents (Bio-Rad; #1705061, Hercules, CA, USA) were added to PVDF membranes (at a 1:1 ratio) and the chemiluminescent signals were imaged by a ChemiDoc MP imaging system (Bio-Rad, Hercules, CA, USA) according to the manufacturer’s specifications. Band densities were then analyzed with ImageJ (the National Institutes of Health, Bethesda, MA, USA), normalized to loading control bands (GAPDH), and then quantified in GraphPad Prism 7 (GraphPad Software, Boston, MA, USA).

### 4.5. Xenograft Experiment

All animal experiments were approved by the Albany Medical College’s Institutional Animal Care and Use Committee. Six-week-old female NCr nude mice (Taconic Biosciences, Germantown, NY, USA) were injected subcutaneously in both flanks with 5,000,000 A2780CIS cells. When palpable tumors formed (ranging from 0.3 cm^3^ to 0.5 cm^3^, as measured by calipers) 12 to 18 days later, mice were evenly distributed across five treatment groups: (1) vehicle, (2) cisplatin, (3) cisplatin + ivermectin, (4) cisplatin + perhexiline, and (5) cisplatin + ivermectin + perhexiline. Cisplatin was administered at 3 mg/kg twice a week, while ivermectin and perhexiline were administered three times a week at 3 mg/kg and 8 mg/kg, respectively. Cisplatin was diluted and administered in normal saline (200 µL per injection), while ivermectin and perhexiline were diluted and administered in DMSO (50 µL per injection). Vehicle treatments were given where appropriate. All vehicle and drug treatments were administered through intraperitoneal (I.P.) injections. Mice in all treatment groups were treated for 2 weeks then euthanized by CO_2_ inhalation followed by cervical dislocation. The size of tumors was measured over the 2-week treatment period. Tumors were collected, weighed, and then processed for immunoblotting and immunofluorescent analysis.

### 4.6. Immunohistochemistry

Formalin-fixed, paraffin-embedded xenograft tumors were cut into 3 µm sections using a microtome (Leica Biosystems, Wetzlar, Germany) by the histology core (Department of Pathology and Laboratory Medicine, Albany Medical College, Albany, NY, USA). Tumor sections were deparaffinized by gentle heating and then with two washes of xylene for 3–5 min each, followed by tissue hydration using a 100, 95, 70, and 50% graded ethanol series for 3–5 min each. Sections were rinsed in PBS twice for 5 min, and then rinsed in sodium citrate buffer (Ph 6) at 95 °C for 20 min. Slides were washed with PBS twice for 5 min each, then permeabilized with PBS + 0.5% Triton-X, washed again, and blocked for 60 min at room temperature using 10% goat serum in PBS. Tissue sections were then incubated overnight at 4 °C with rabbit anti-HSP27 (1:250; Abcam; #ab32501, Cambridge, UK) and mouse anti-CPT1A (1:250; Abcam; #ab128568, Cambridge, UK) primary antibodies diluted in wash buffer containing 2% BSA, followed by brief PBS wash and then incubation with goat anti-rabbit (Alexa Fluor 594) or goat anti-mouse secondary (FITC-conjugated) (1:500; Invitrogen; #31569 and A11037, Waltham, MA, USA) incubation for 1 h at room temperature. After washing with PBS (3 times, 5 min each), slides were incubated with blocking buffer with DAPI (1:5000; ThermoFischer, #62248, Waltham, MA, USA) for 15 min. After DAPI incubation and 3 more PBS washes, coverslips were mounted using Prolong anti-fade gold mounting medium (Invitrogen; #P36930, Waltham, MA, USA). Images were acquired at 40× magnification on an Olympus BX61 upright microscope with a PCO.EDGE 4.2 scientific CMOS camera (Evident Scientific; Waltham, MA, USA). A list of antibodies and other drugs and reagents are listed in Table S1.

### 4.7. Flow Cytometry

A2780CIS or PEO4 cells were cultured in either unstimulated conditions or treated with monotherapies or combinations of saline (vehicle), 16 µM cisplatin (CIS), or 1.5 µM ivermectin (IVM) for 36 h. Live A2780CIS or PEO4 cells were collected and stained with Tetramethyl rhodamine, ethyl ester (TMRE; Abcam; #AB113852, Cambridge, UK), MitoTracker (ThermoFisher # M7514, Waltham, MA, USA), MitoSOX (ThermoFisher # M36008, Waltham, MA, USA), CellROX (ThermoFisher #C10444, Waltham, MA, USA), ThiolTracker (ThermoFisher # T10095, Waltham, MA, USA), or Image—iT Lipid Peroxidation kit (ThermoFisher #C10445, Waltham, MA, USA) in flow buffer (PBS supplemented with 1% BSA and 2 mM EDTA) for 10–30 min at room temperature, according to the manufacturers’ recommendations. For the Image—iT Lipid Peroxidation kit (ThermoFisher #C10445, Waltham, MA, USA), lipid peroxidation was measured through ratio analysis of 510 nM to 590 nM emission fluorescence from the kit’s dye, as per manufacturer’s instructions. Cells were then washed with flow buffer followed by acquisition with the ZE5 flow cytometer cell analyzer (Bio-Rad, Hercules, CA, USA) or the FACSymphony flow cytometer cell analyzer (BD Biosciences, San Jose, CA, USA). 500,000 events were analyzed per group using FlowJo software (version 10.4.2; BD Biosciences, San Jose, CA, USA).

### 4.8. Statistical Analysis

Statistical analyses were performed using GraphPad Prism software version 7.0 (GraphPad Software, Boston, MA, USA). A Student’s *t*-test (unpaired two-tailed two-sample or one-sample) was used to compare the means of two groups, and one-way ANOVA analysis was used to compare the mean difference of three or more groups. For the analysis of tumor growth over time, line slopes were compared via one-way ANOVA. *p* ≤ 0.05 was considered significant.

## 5. Conclusions

Our results show that cisplatin-resistant ovarian cancer cell lines upregulate HSP27 and FAO to deplete cisplatin-induced ROS to attenuate cisplatin’s cytotoxic effect. Dual inhibition of HSP27 and FAO is a promising therapeutic strategy for cisplatin-resistant ovarian cancer.

## Data Availability

All data and reagents in this publication can be shared upon request to the PI.

## Supplementary Materials

The following supporting information can be downloaded at: https://www.mdpi.com/article/10.3390/ijms241612638/s1.
