# Supplementary material for: Targeting Heat Shock Protein 27 and Fatty Acid Oxidation Augments Cisplatin Treatment in Cisplatin-Resistant Ovarian Cancer Cell Lines
Source: Int J Mol Sci. 2023 Aug 10;24(16):12638. doi: 10.3390/ijms241612638 (PMC10454186; doi:10.3390/ijms241612638)
Supplement: Supplementary file 1 [file ijms-24-12638-s001.zip › ijms-2501187-SI/HSP27 and FAO manuscript supplementary figures_final_8.9.23.pdf]

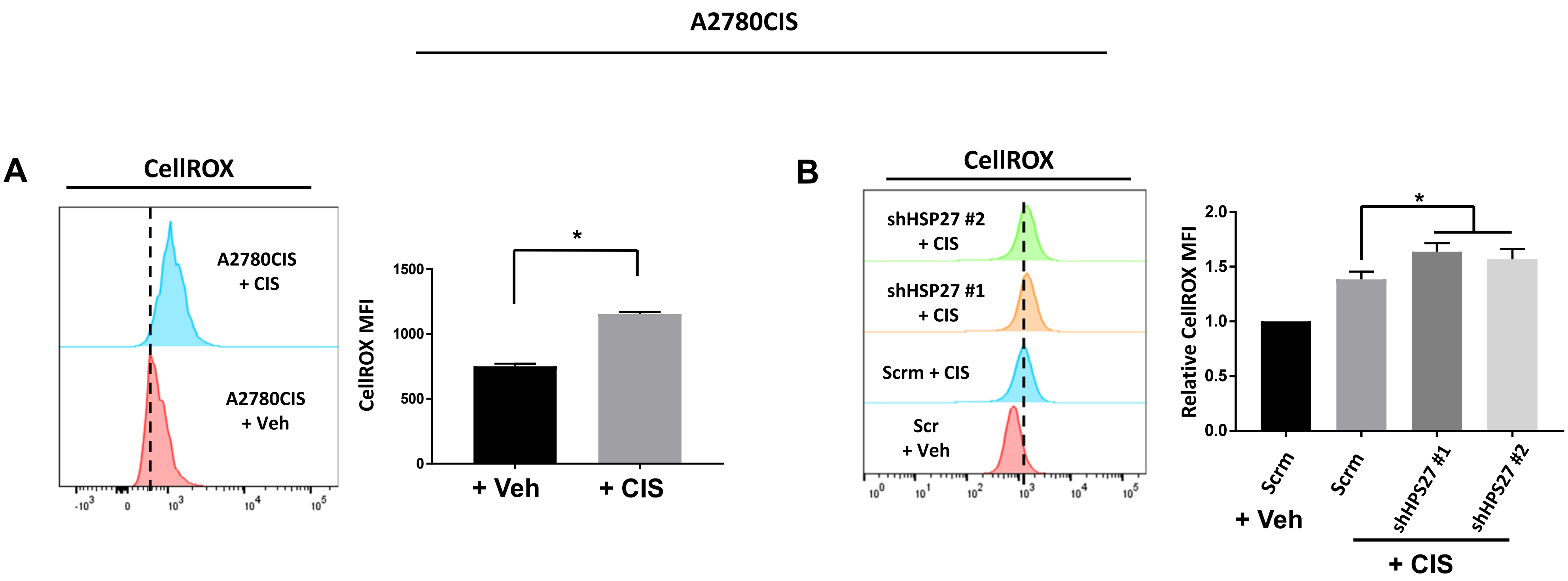

Figure S1. HSP27 knockdown increases cisplatin induces cell ROS in A2780CIS cisplatin-resistant ovarian cancer cells
(A) Flow cytometric analysis of A2780CIS cells treated with vehicle (Veh) treatment or 16 µM Cisplatin (CIS) assessed with CellROX. (B)) Flow cytometric analysis of A2780CIS scramble control and knockdown cells treated with vehicle (Veh) treatment or 16 µM Cisplatin (CIS) assessed with CellROX. Unpaired two-tailed 2-sample t-test or one-way ANOVA. Data are reported as mean ± SD. N=3. P-value of <0.05 = *.

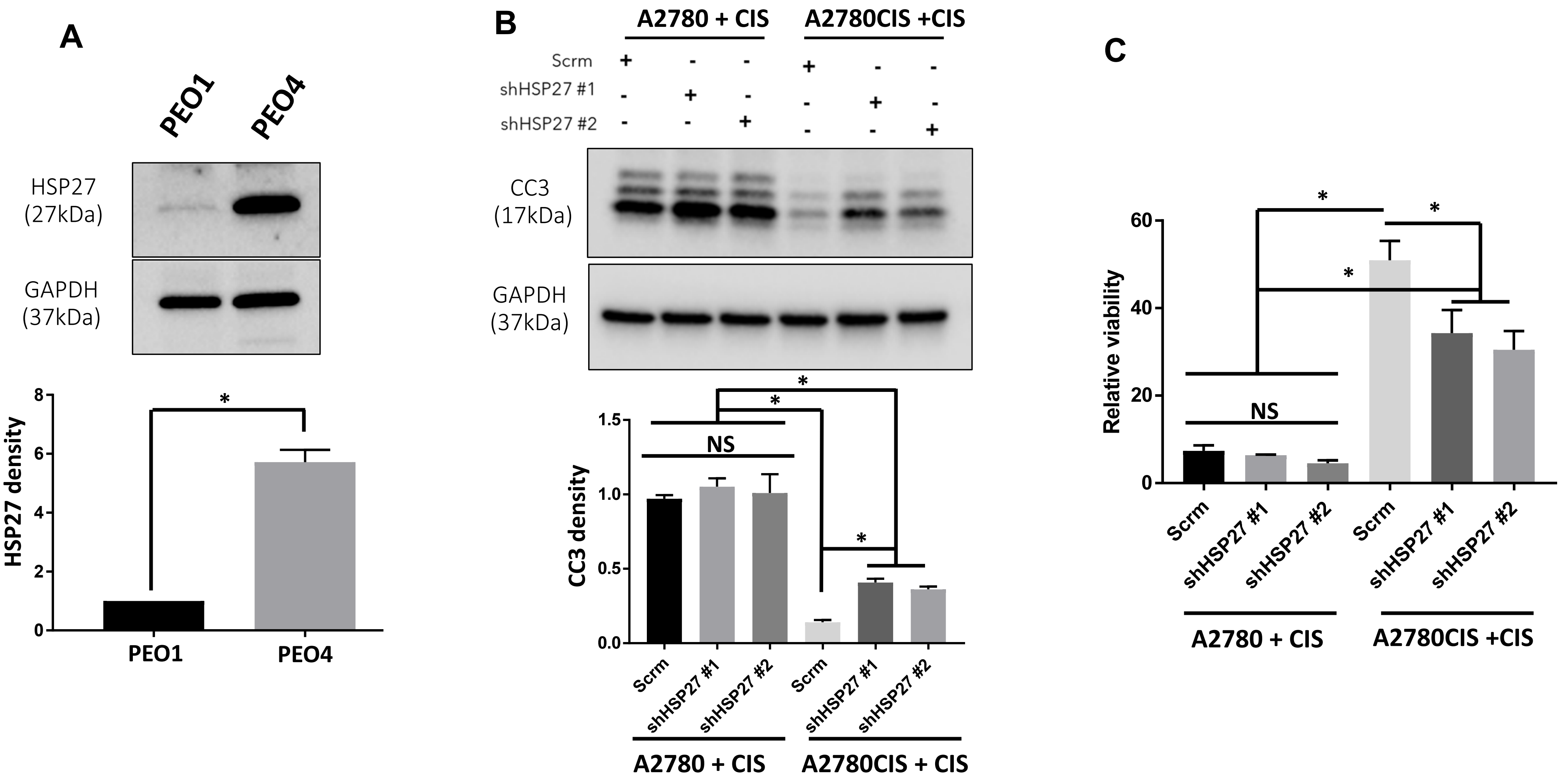

Figure S2. HSP27 is highly expressed in cisplatin resistant ovarian cancer cells and HSP27 knockdown efficiency

(A) PEO1 and PEO4 cells assessed for expression of HSP27. (B) A2780 and A2780CIS scrambled control (scrm) and HSP27 knockdown cells assessed for cleaved caspase-3 (CC3). Quantifications of HSP27 and CC3 (normalized to GAPDH) from 3 experiments (N=3) in bar graphs below blots. Unpaired two-tailed 2-sample t-test or one-way ANOVA. (C) Relative cell viability of A2780 and A2780CIS (measured with MTT kit) scramble control and HSP27 knockdown cells treated with 16 μM Cisplatin (CIS). Data are reported as mean ± SD. P-value of <0.05 = *. Uncropped western blot images are displayed in Figure S12.

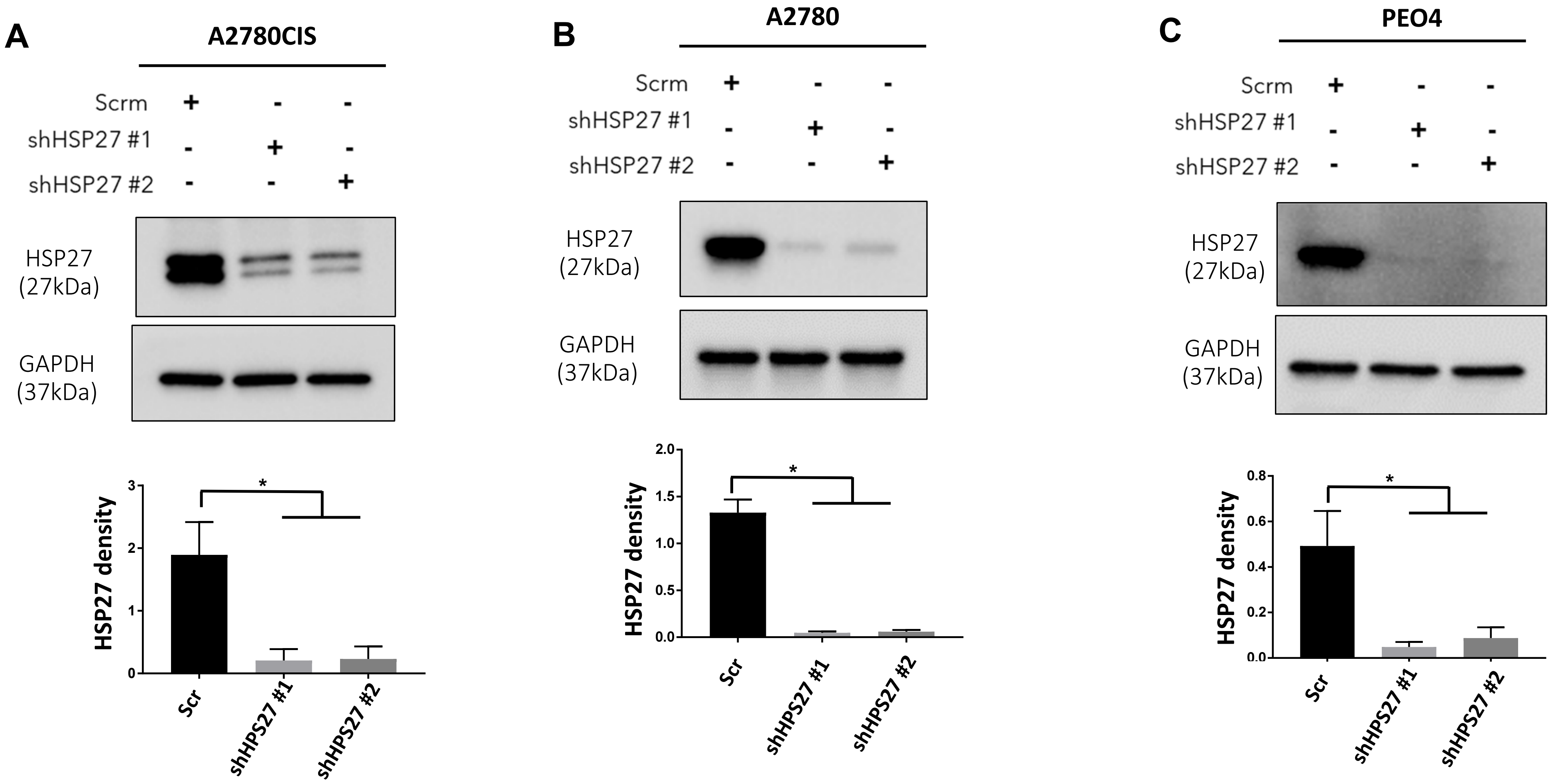

Figure S3. HSP27 knockdown efficiency in ovarian cancer cell lines
(A-C) A2780CIS (A), A2780 (B) and PEO4 (C) scrambled control (scrm) and knockdown cells assessed for HSP27. Quantifications of HSP27 (normalized to GAPDH) from 3 experiments (N=3) in bar graphs below blots. One-way ANOVA. Data are reported as mean ± SD. P-value of <0.05 = *. Uncropped western blot images are displayed in Figure S13.

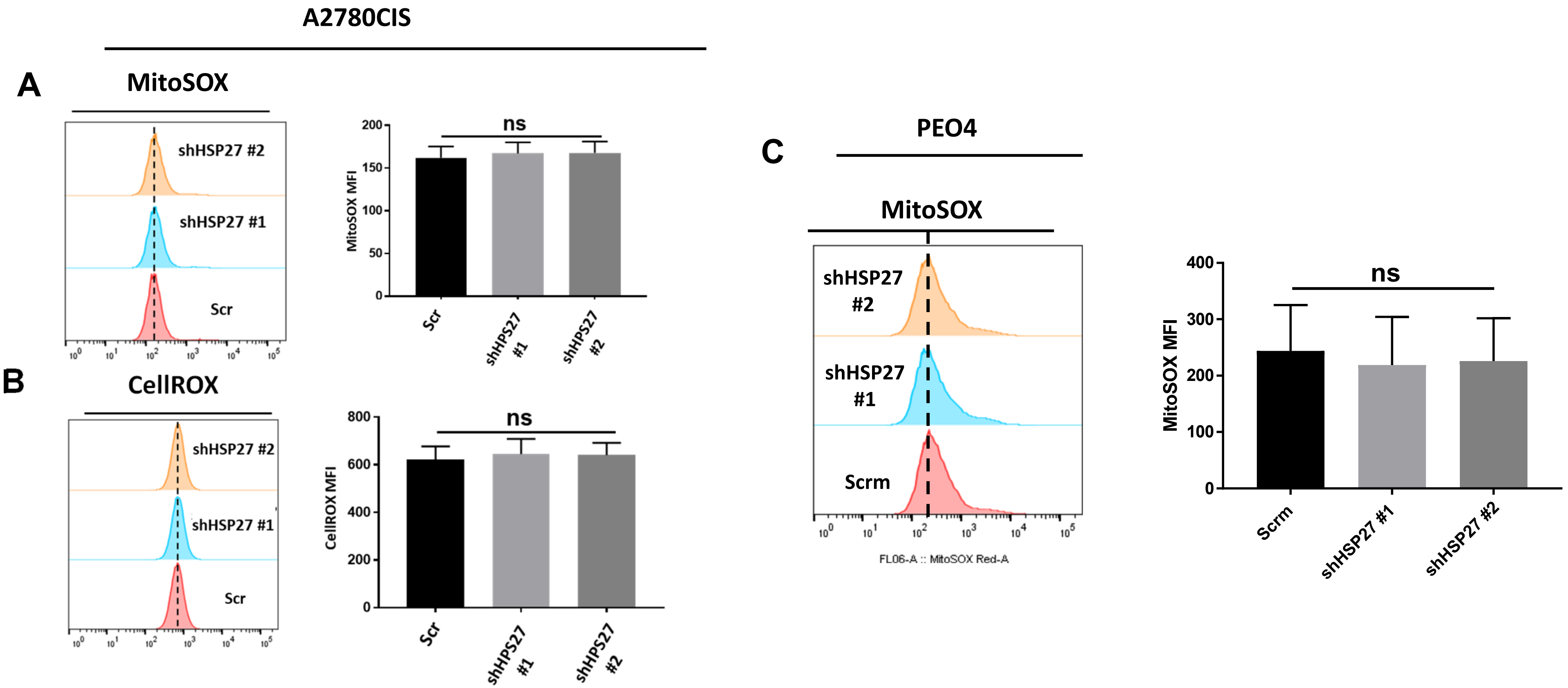

Figure S4. Basal levels of cellular and mitochondtial ROS in control and HSP27 knockdown cells
(A-B) Flow cytometric analysis of A2780CIS control and HSP27 knockdown assessed with MitoSOX (A) and CellROX (B). (C) PEO4 control and HSP27 knockdown cells assessed with MitoSOX. Quantifications of 3 experiments (N=3) shown in graphs to the right. One-way ANOVA. Data are reported as mean ± SD. P-value of <0.05 = *.

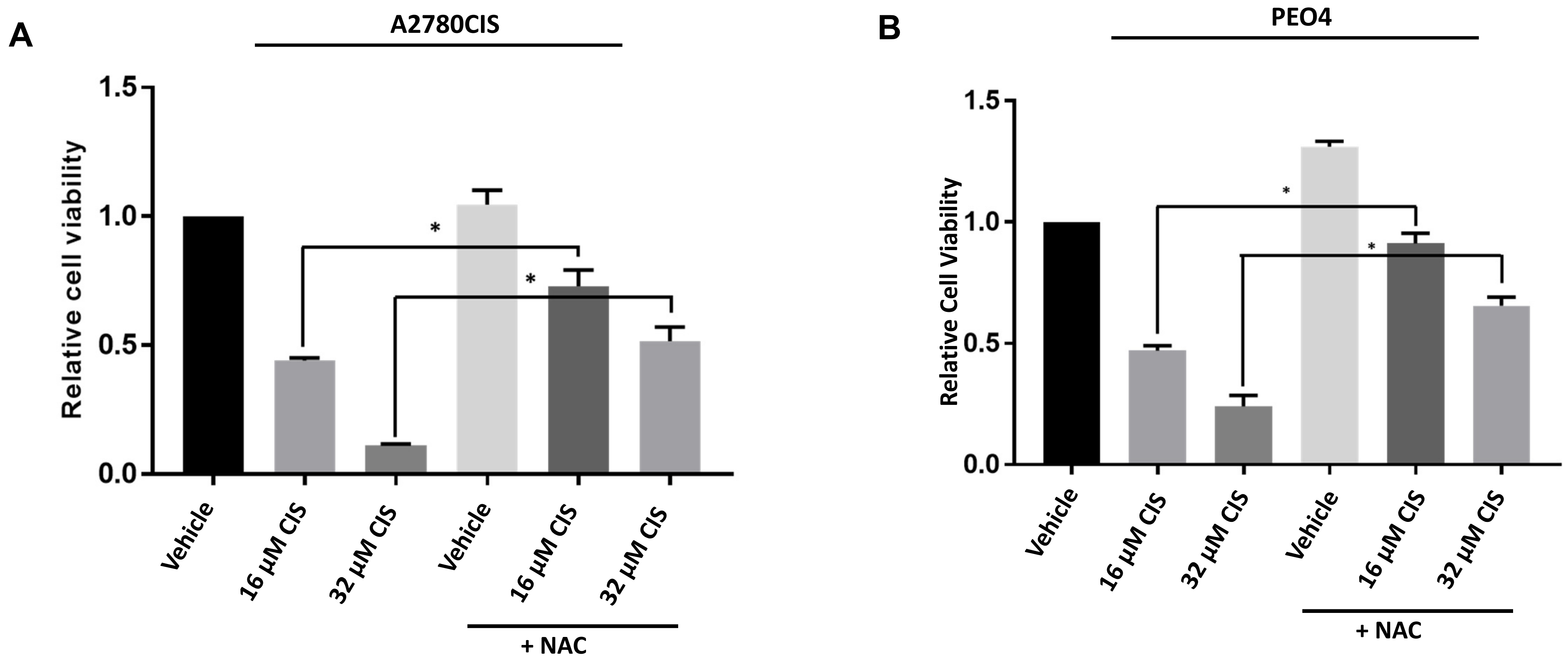

Figure S5. ROS depletion though N-Acetyl-Cysteine (NAC) attenuates cisplatin induced loss of cell viability (A-B) Relative cell viability of A2780CIS (A- measured with acid phosphatase kit) and PEO4 (B- measured with MTT kit) cells treated with vehicle (Veh) treatment or 16 µM, or 32 µM Cisplatin (CIS) with and without 2.5mM N-Acetyl-Cysteine (NAC). One-way ANOVA. Data are reported as mean ± SD. N=3. P-value of <0.05 = *.

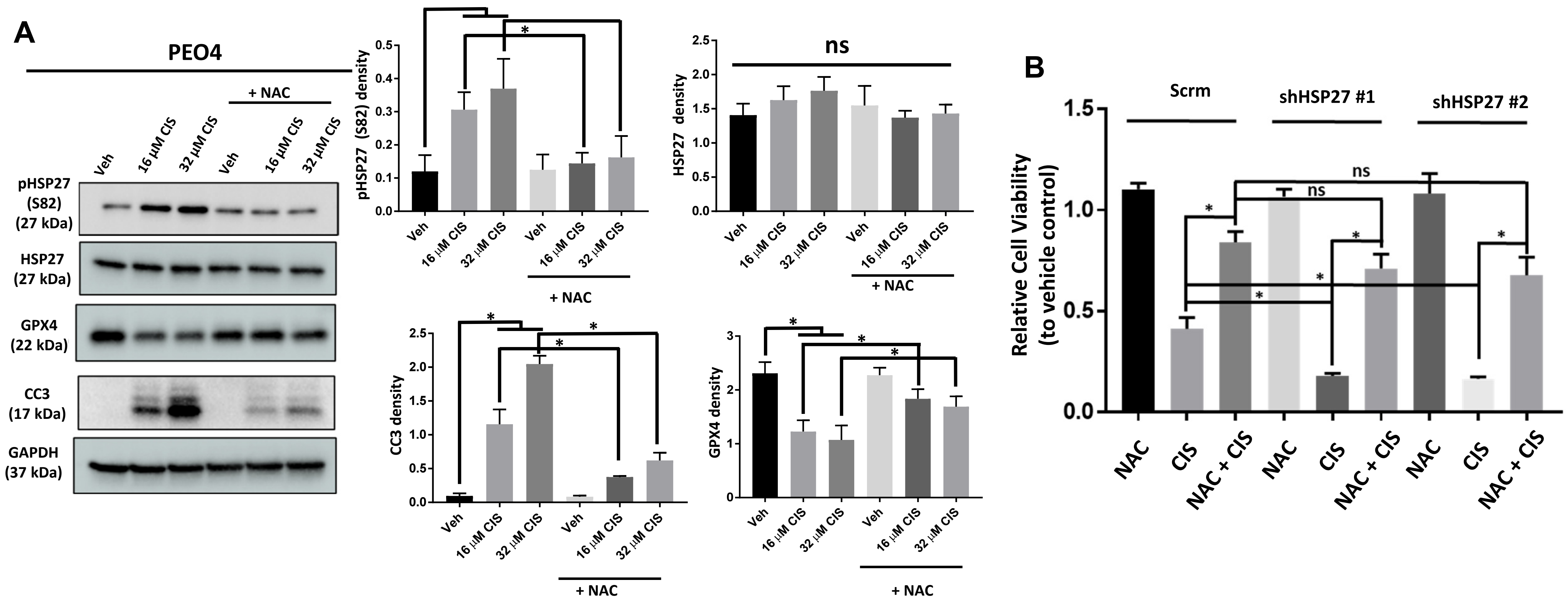

Figure S6. ROS depletion though N-Acetyl-Cysteine (NAC) attenuates cisplatin induced HSP27 and cell death and rescues loss of HSP27 knockdown cell viability (A) Western blot of total phosphorylated HSP27, total HSP27 GPX4, or cleaved caspase-3 (CC3) in PEO4 cells treated with DMSO (Veh), 16 or 32 µM Cispaltin (CIS), 2.5mM N-Acetyl Cysteine (NAC), or combination (CIS+NAC). Quantification of Western blot markers (normalized to GAPDH) quantified to graphs on the right. (B) Relative cell viability (measured by MTT assay) of A2780CIS-scrm or A2780CIS-shHSP27 cells treated with 2.5 mM N-Acetyl Cysteine (NAC), 16 µM cisplatin (CIS), or combination (NAC + CIS) compared to respective vehicle controls. One-way ANOVA. Mean ± SD. *, p value <0.05. N=3. Uncropped western blot images are displayed in Figure S14.

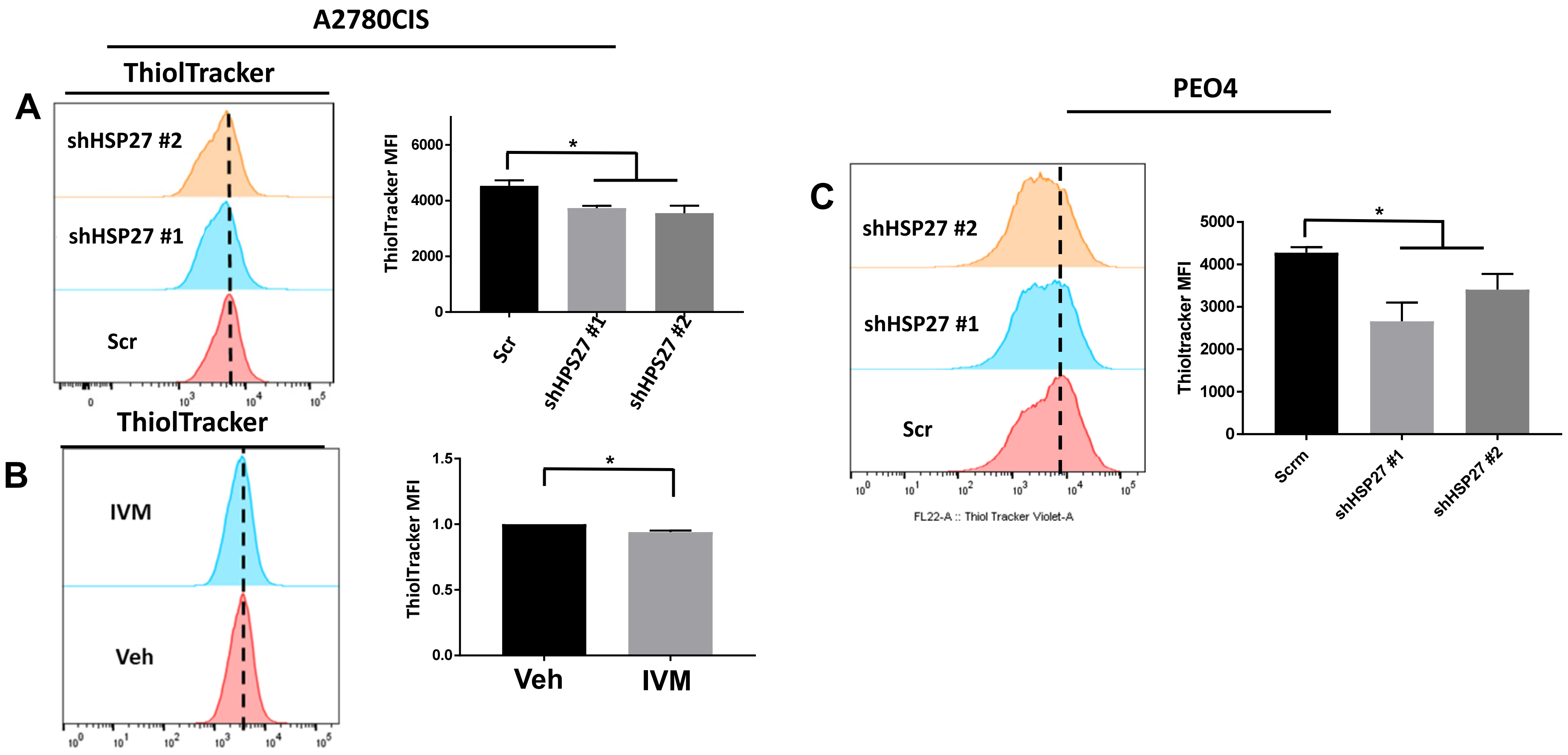

Figure S7. Basal levels of reduced thiols (GSH) in control and HSP27 knockdown and inhibited cells
(A) Flow cytometric analysis of A2780CIS control and HSP27 knockdown assessed with ThiolTracker (B) A2780CIS cells treated with vehicle (Veh) or ivermectin treatments assessed with ThiolTracker. (C) PEO4 control and HSP27 knockdown cells assessed with ThiolTracker. Quantifications of 3 experiments (N=3) shown in graphs to the right. One-way ANOVA. Data are reported as mean ± SD. P-value of <0.05 = *.

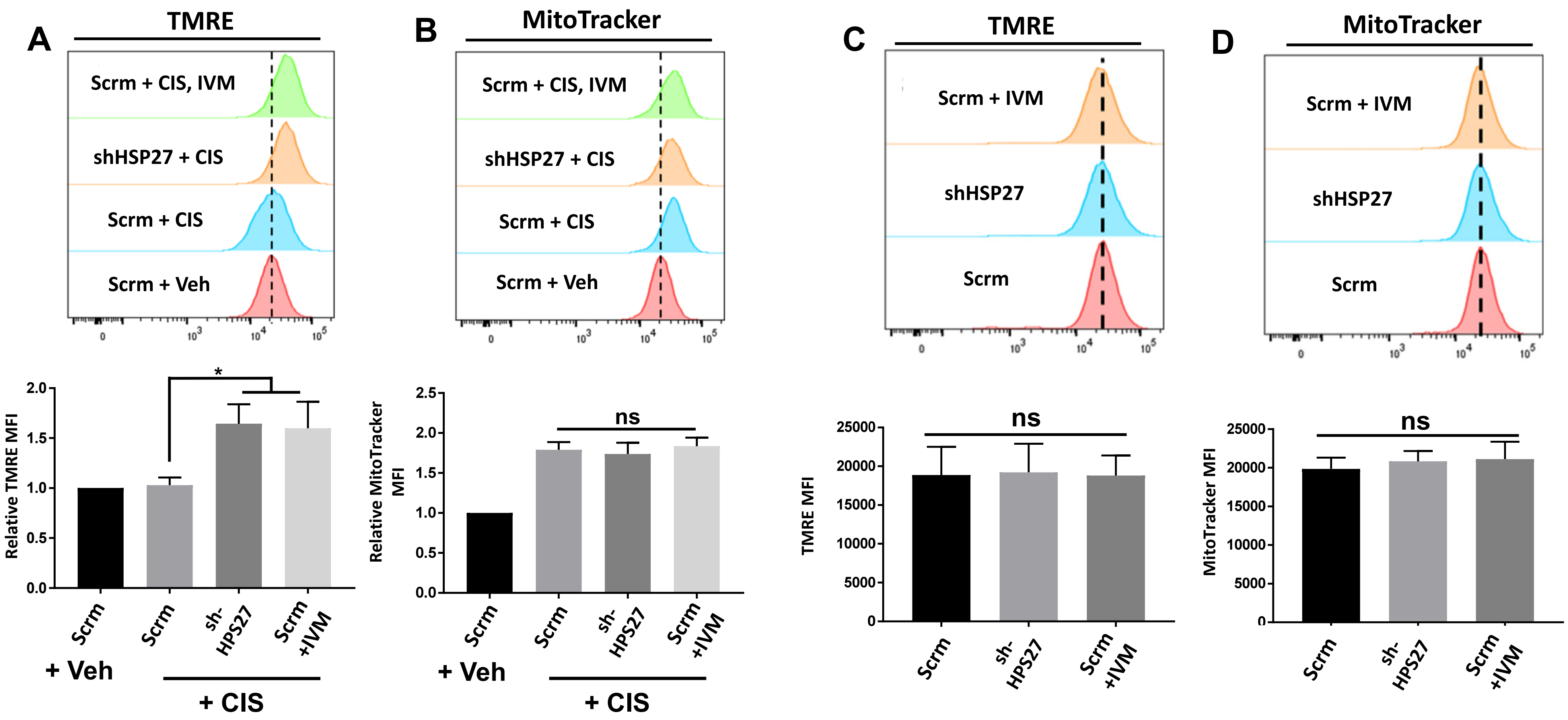

Figure S8. HSP27 inhibition increases mitochondrial membrane potential after cisplatin treatment
(A-B) Flow cytometric analysis of TMRE (tetramethylrhodamine, ethyl ester) (A) or Mitotracker staining (B) in A2780CIS-scramble (scr) or A2780CIS-shHSP27 cells treated with DMSO (Veh), 16 μM cisplatin (CIS), or drug combination (1.5 μM ivermectin (IVM) + 16 μM cisplatin). (C-D) Flow cytometric analysis of TMRE (tetramethylrhodamine, ethyl ester) (D) or Mitotracker staining (D) in A2780CIS-scramble (scr) or A2780CIS-shHSP27 cells treated with DMSO (Veh), or μM ivermectin (IVM). One-way ANOVA. Mean ± SD. *, p value <0.05. NS, not significant. N=3.

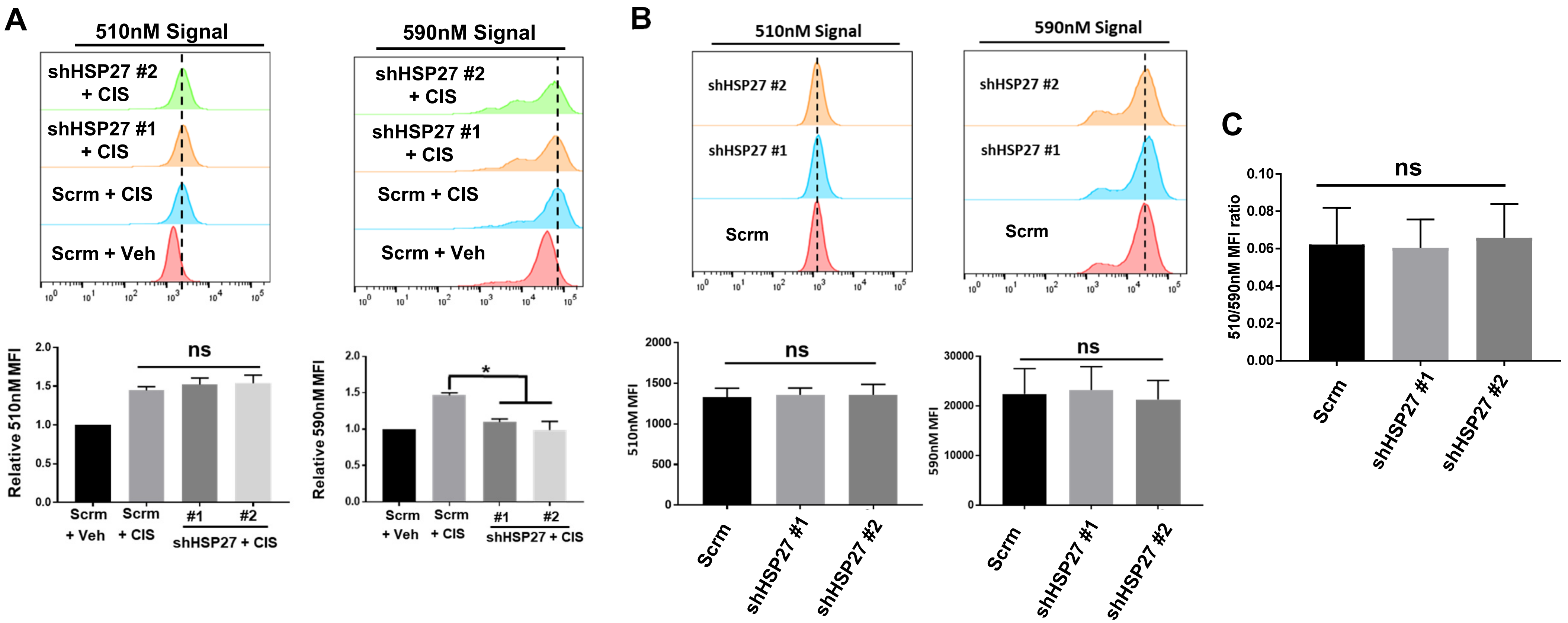

Figure S9. Levels of 510nM and 590nM signals from Image iT peroxidation dye signal in control and HSP27 knockdown cells, with and without cisplatin treatment

(A) Flow cytometry of scramble control or HSP27-knockdown A2780CIS cells treated with vehicle (Veh) or 16 µM Cisplatin (CIS) and then assessed with Image-iT Lipid peroxidation dye. Flow plots show individual emission of Image iT peroxidation dye (at 510nM and 590nM). (B-C) Flow cytometric analysis of A2780CIS control and HSP27 knockdown assessed with Image iT peroxidation dye assessed for 510nM and 590nM signal and (B) ratio analysis of 510nM/590nM. Quantifications of 3 experiments (N=3) shown in graphs to the right. One-way ANOVA. Data are reported as mean ± SD. P-value of <0.05 = *.

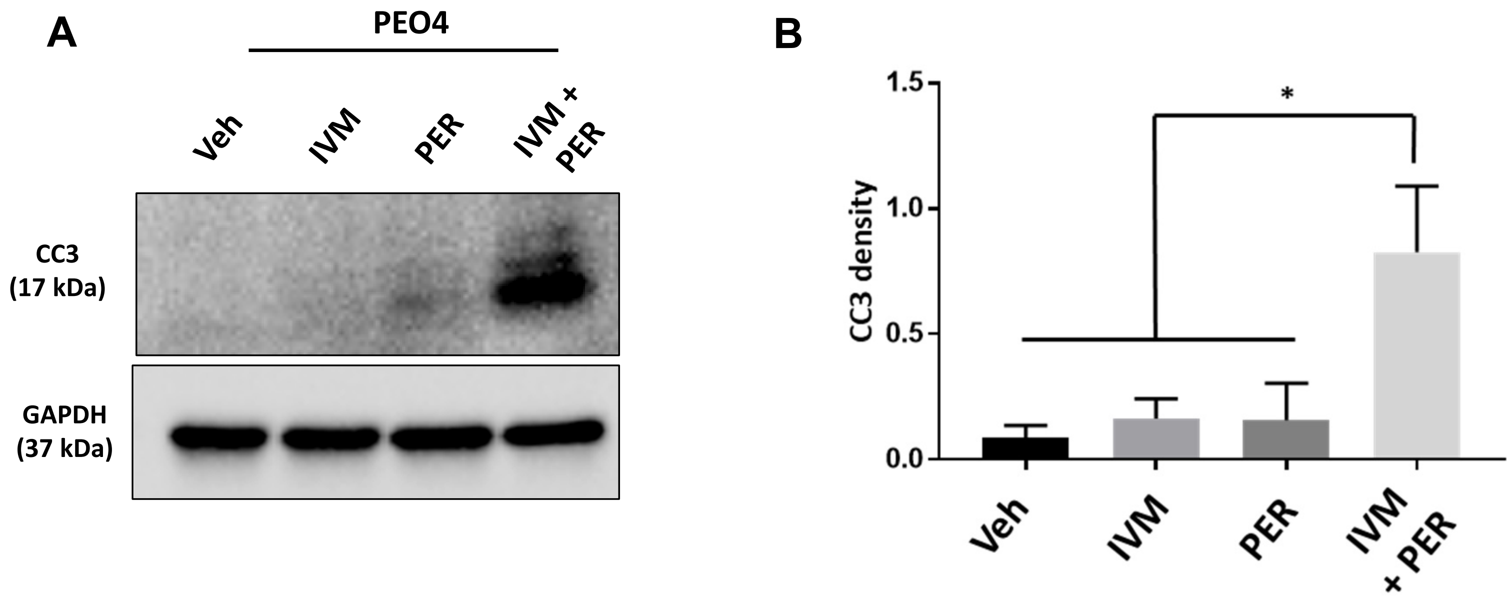

Figure S10. Dual inhibition of HSP27 and FAO (with ivermectin and perhexiline) increases PEO4 cell apoptosis
(A) Western blot of cleaved caspase-3 (CC3) in PEO4 cells treated with DMSO (Veh), 5 μM ivermectin (IVM), 7.5 μM perhexiline (PER), or drug combination (IVM + PER). (B) Quantification of CC3 (normalized to GAPDH) on graph to the right of blot. One-way ANOVA. Mean ± SD. *, p value <0.05. N=3. Uncropped western blot images are displayed in Figure S17.

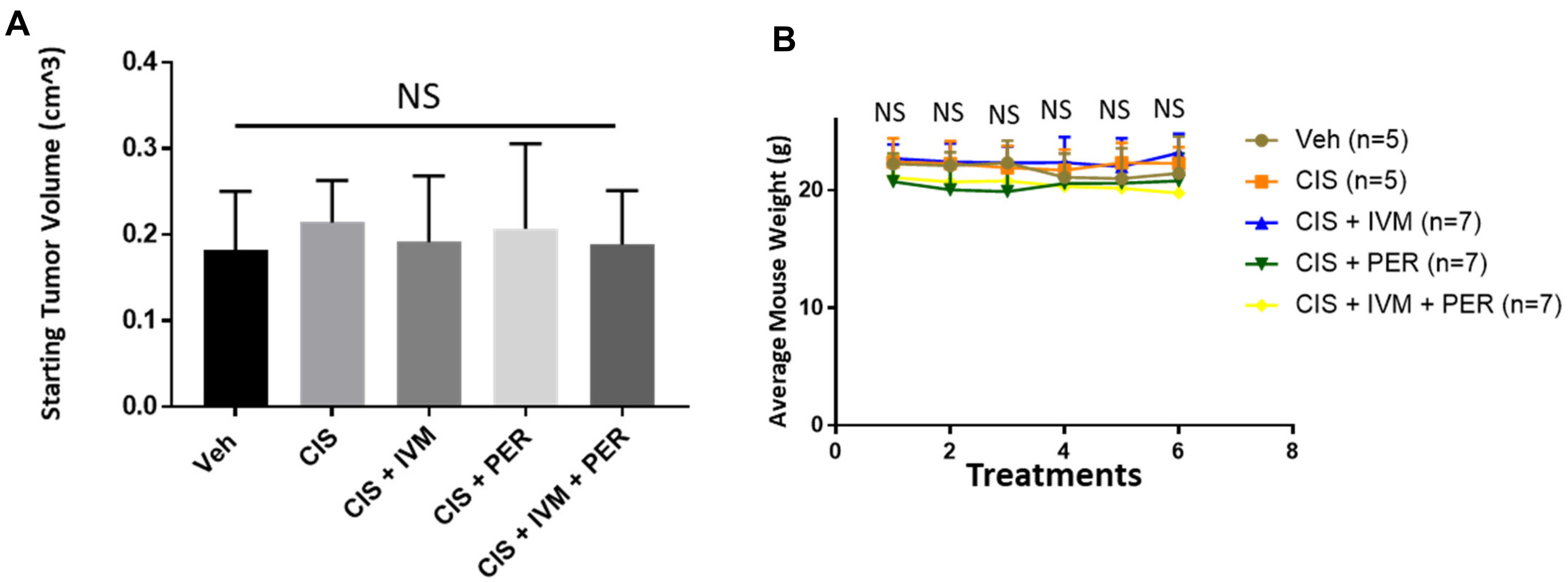

Figure S11. Starting A2780CIS xenograft tumor sizes and mouse weight over time
(A) A2780CIS xenograft tumor volume (in cm^3) measured on the first day of drug treatment. Veh: vehicle. CIS: cisplatin, IVM: ivermectin, PER: perhexiline. (B) Average body weight of mice in each treatment group throughout experimentation. N= indicates number of mice. One-way ANOVA was used in (A) and for each individual treatment time point in (B). Mean ± SD. NS = not significant.

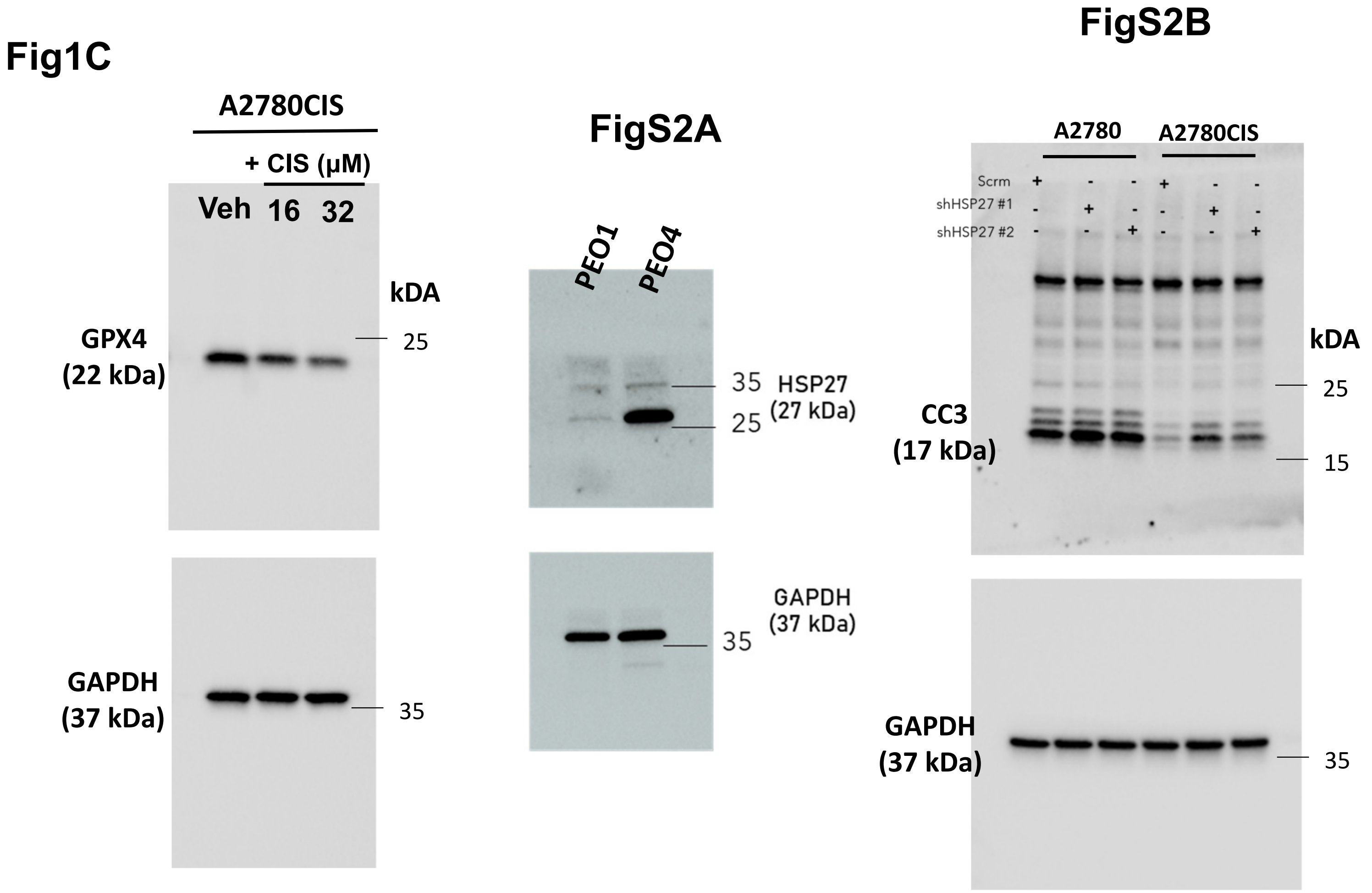

Figure S12. Uncropped western blot images for Fig1C, FigS2A, FigS2B

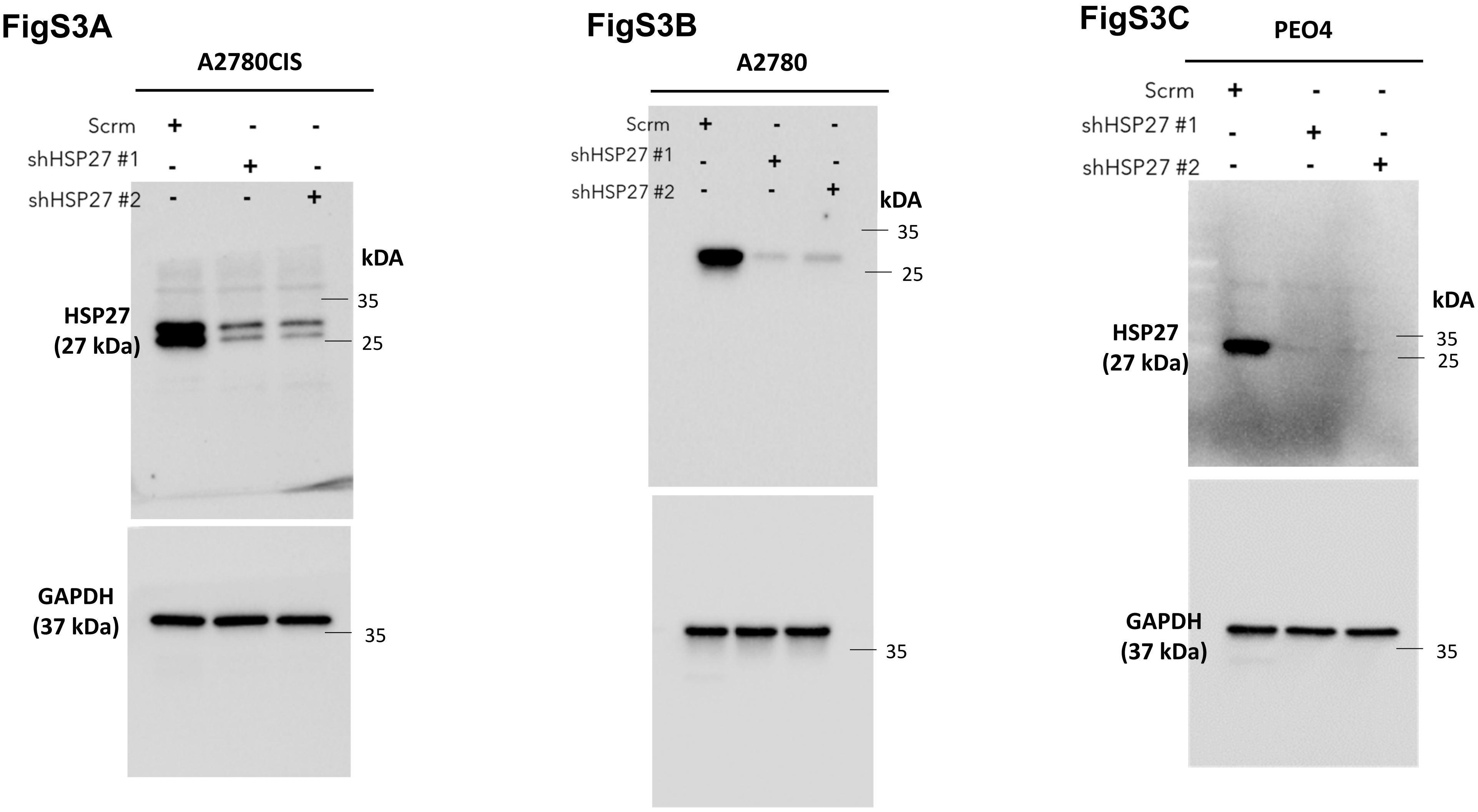

Figure S13. Uncropped western blot images FigS3A, FigS3B, FigS3C

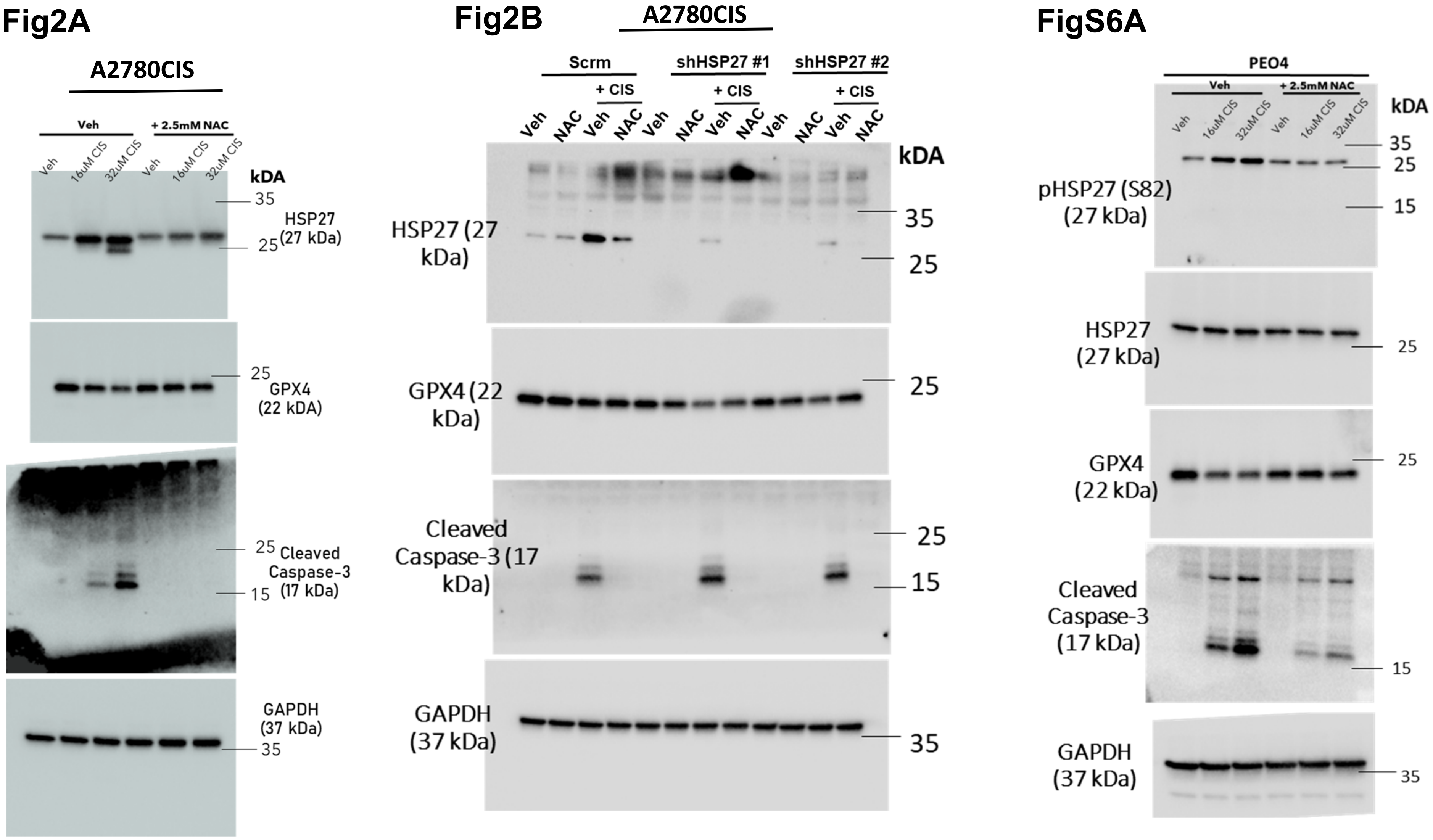

Figure S14. Uncropped western blot images for Fig2A, Fig2B, FigS6A

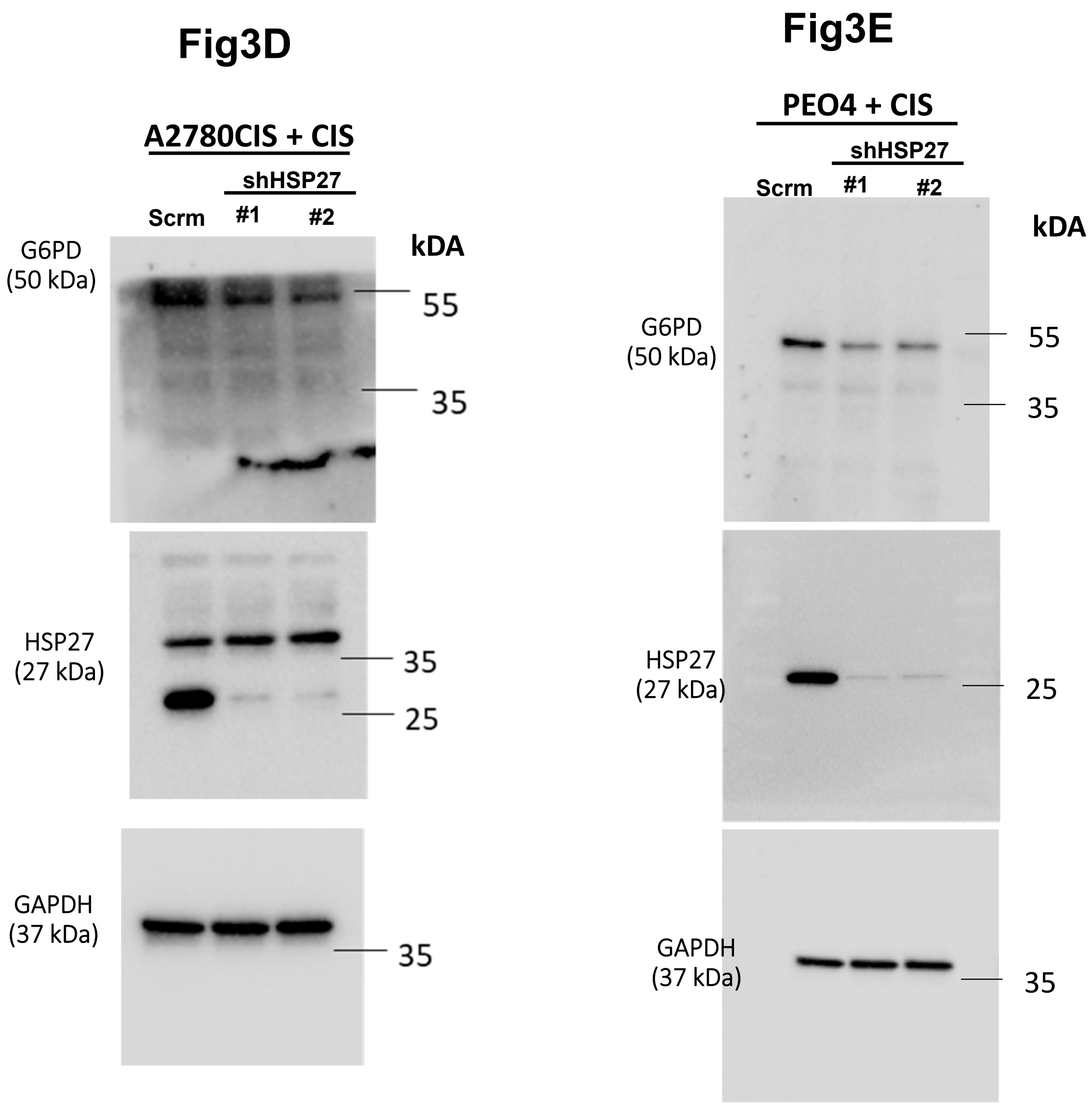

Supplementary Figure 15. Uncropped western blot images for Fig3D, Fig3E

Fig4B

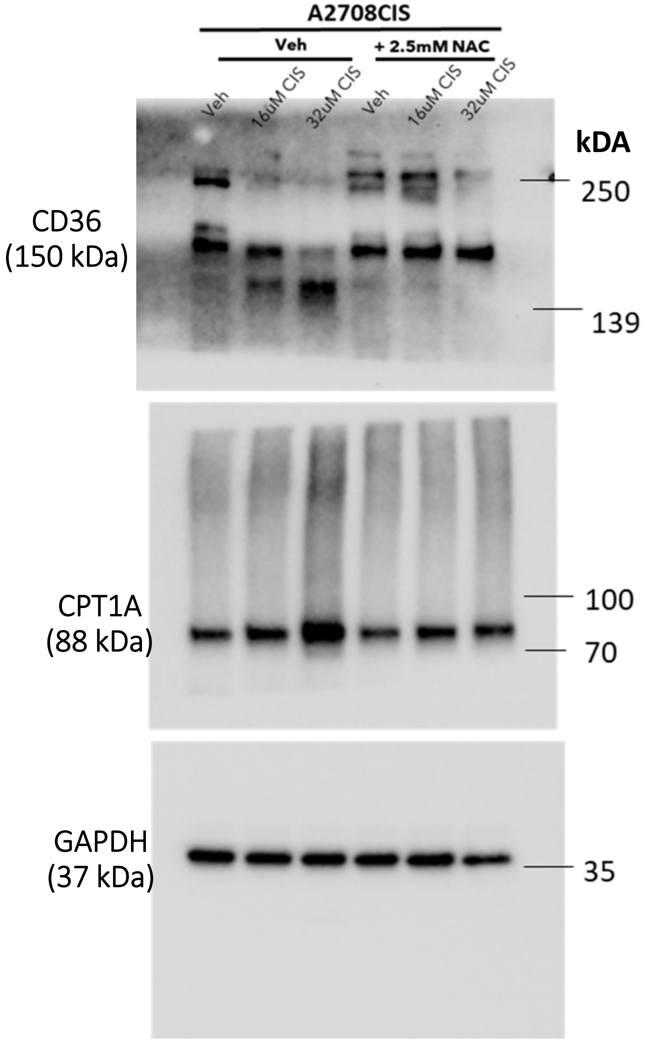

Fig4C

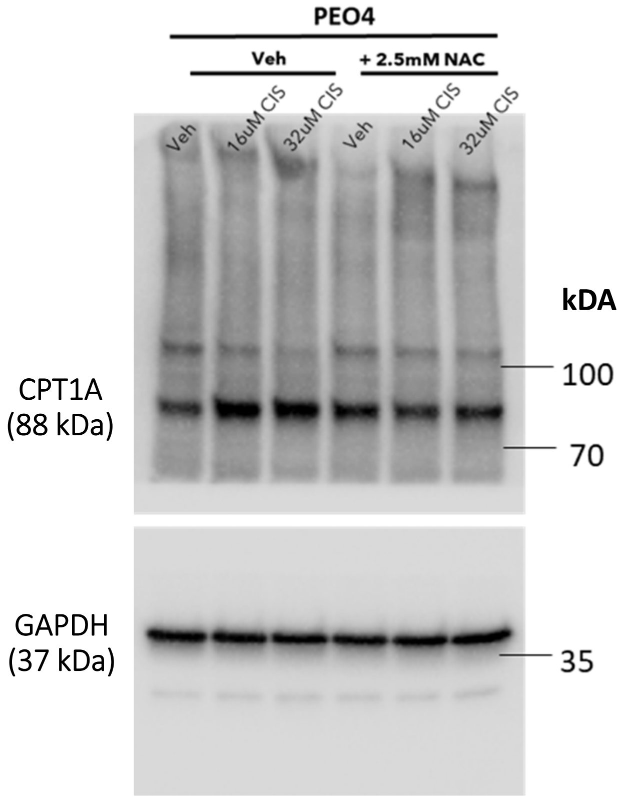

Fig4D

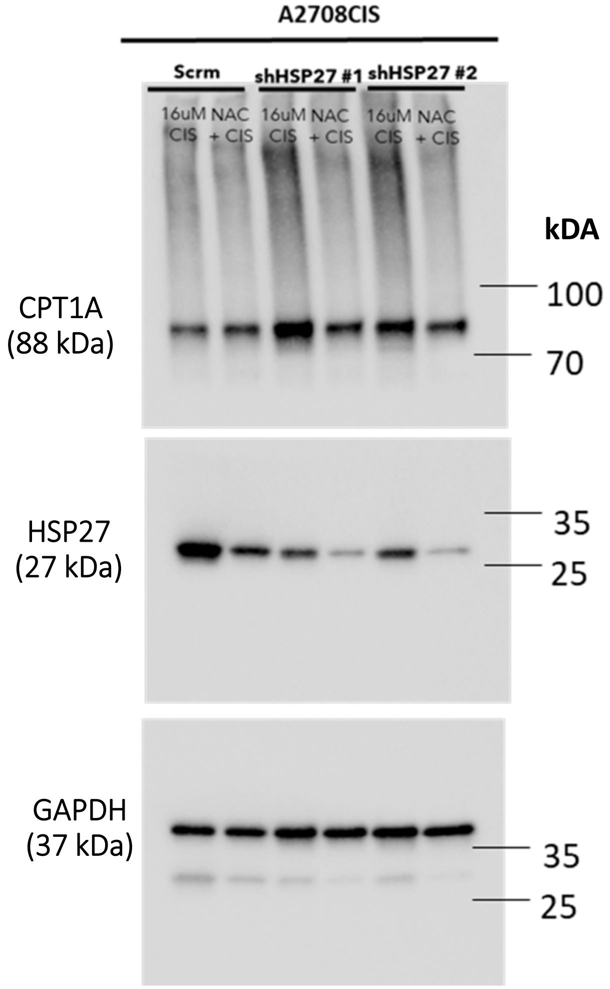

Figure S16. Uncropped western blot images for Fig4B, Fig4C, Fig4D

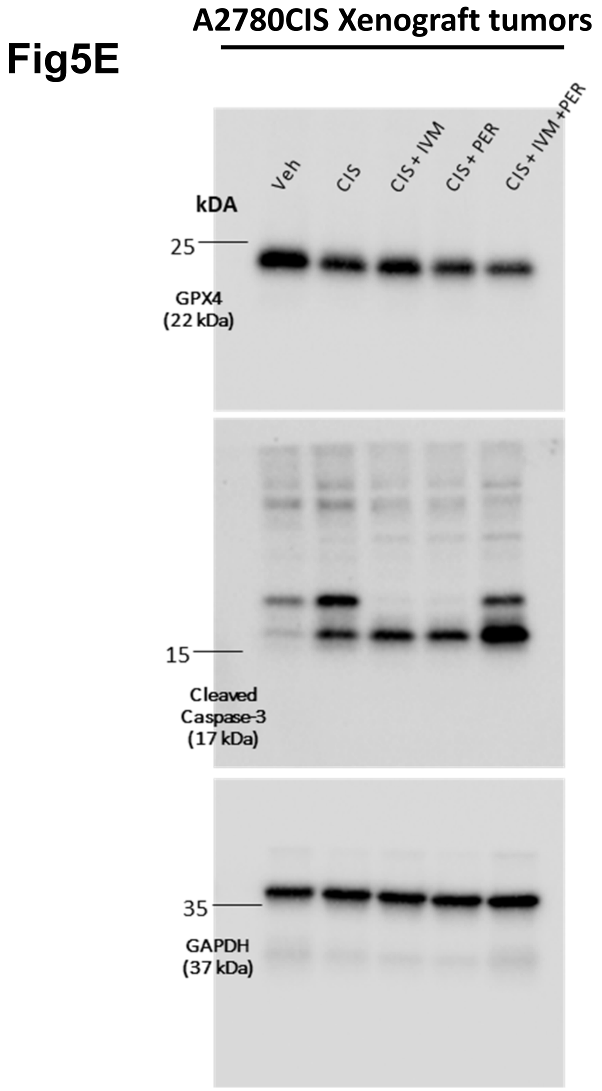

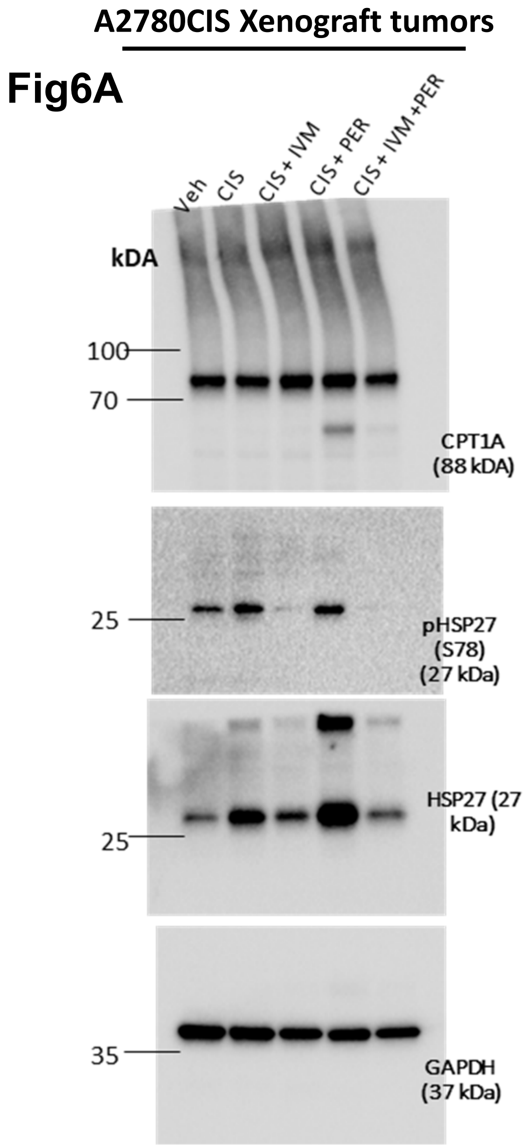

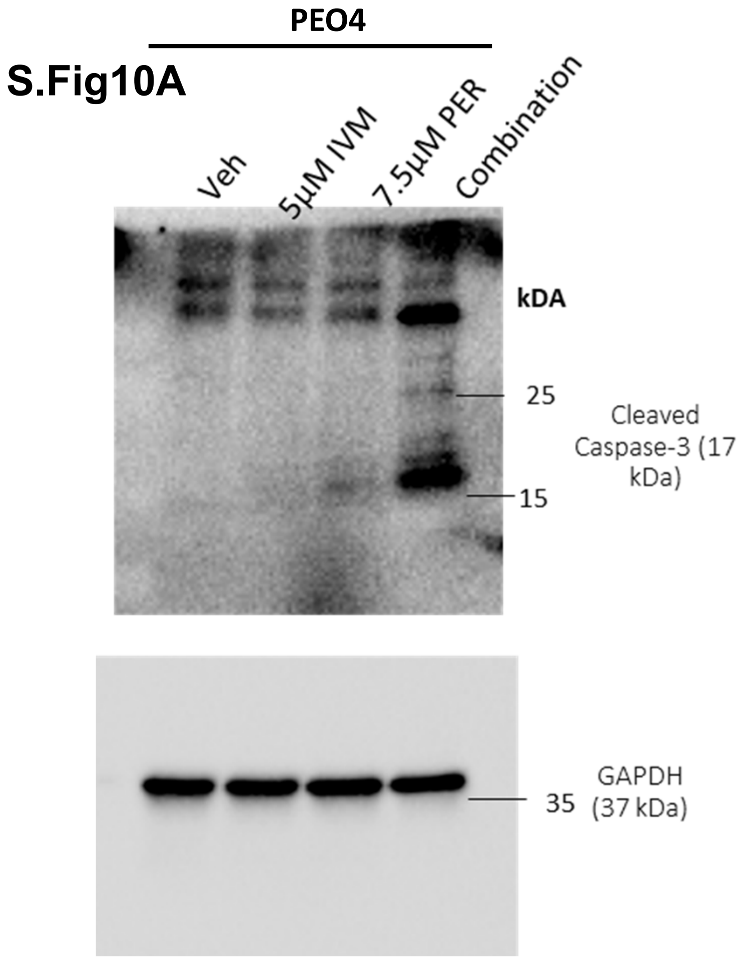

Figure S17. Uncropped western blot images for Fig5E, Fig6A, FigS10A
