# Supplementary material for: Targeting Heat Shock Protein 27 and Fatty Acid Oxidation Augments Cisplatin Treatment in Cisplatin-Resistant Ovarian Cancer Cell Lines
Source: Int J Mol Sci. 2023 Aug 10;24(16):12638. doi: 10.3390/ijms241612638 (PMC10454186; doi:10.3390/ijms241612638)
Supplement: Supplementary file 1 [file ijms-24-12638-s001.zip › ijms-2501187-SI/manuscript raw westerns final.pdf]

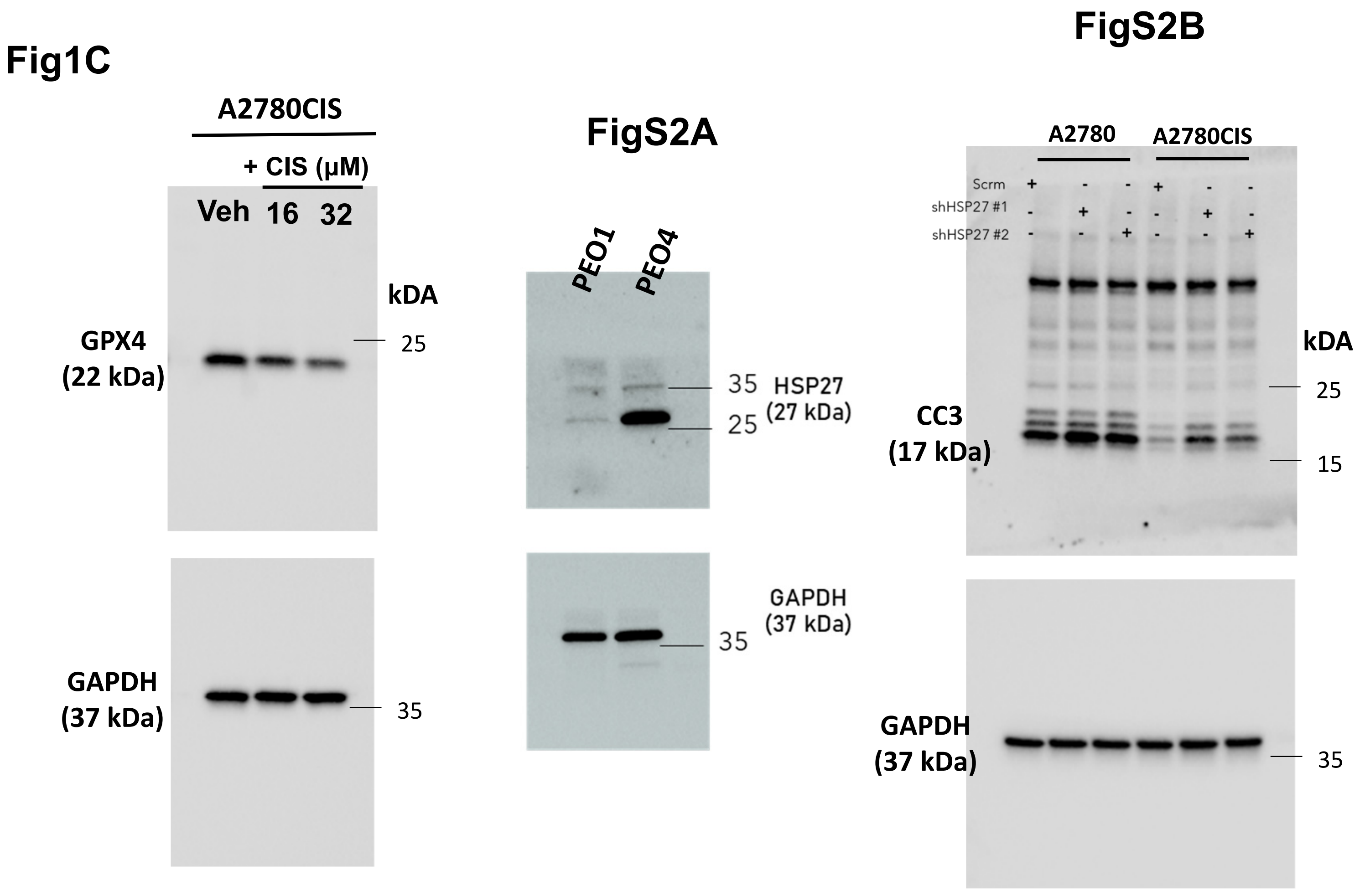


**Figure S12.** Uncropped western blot images for Fig1C, FigS2A, FigS2B

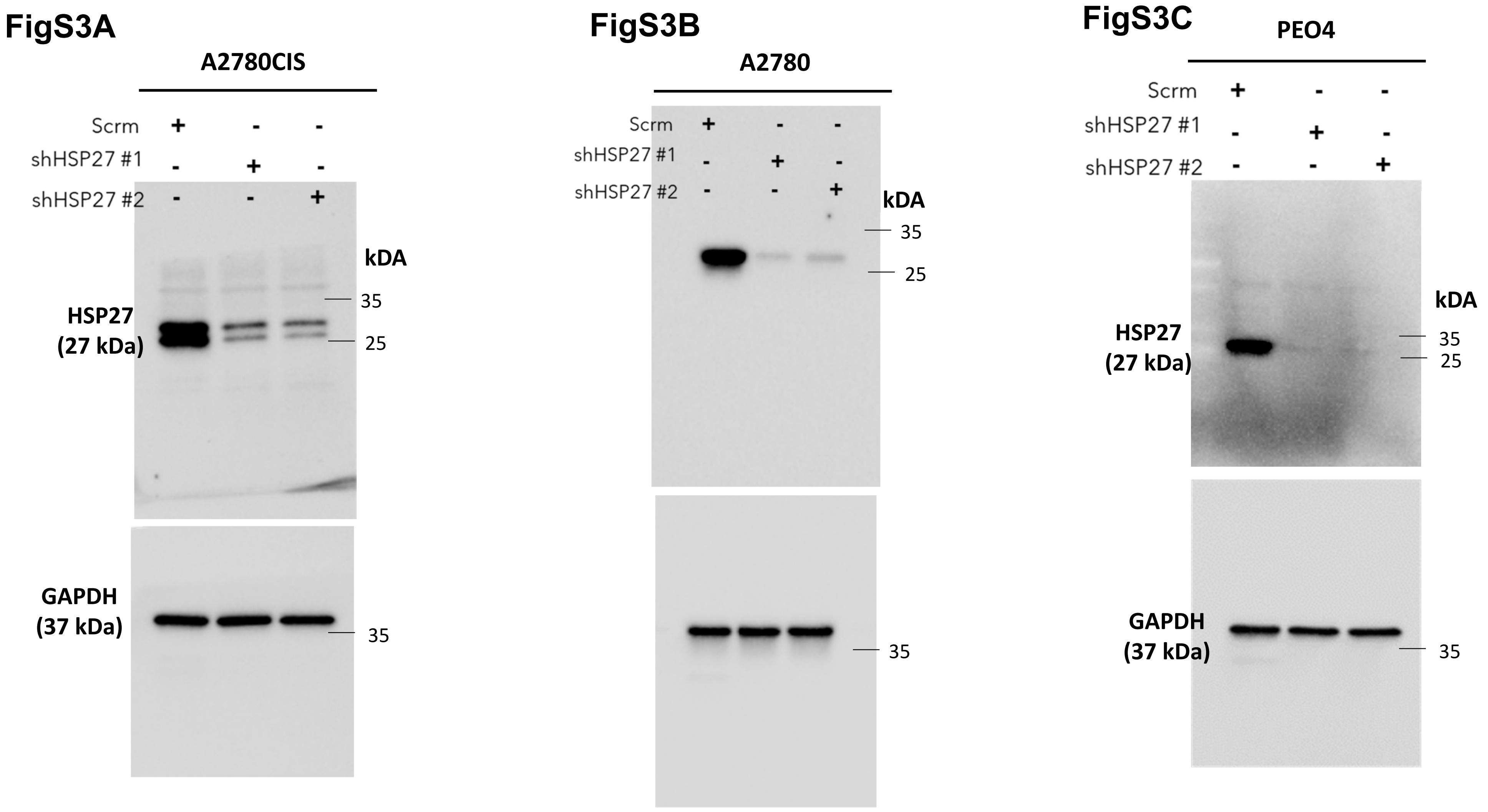


**Figure S13.** Uncropped western blot images FigS3A, FigS3B, FigS3C

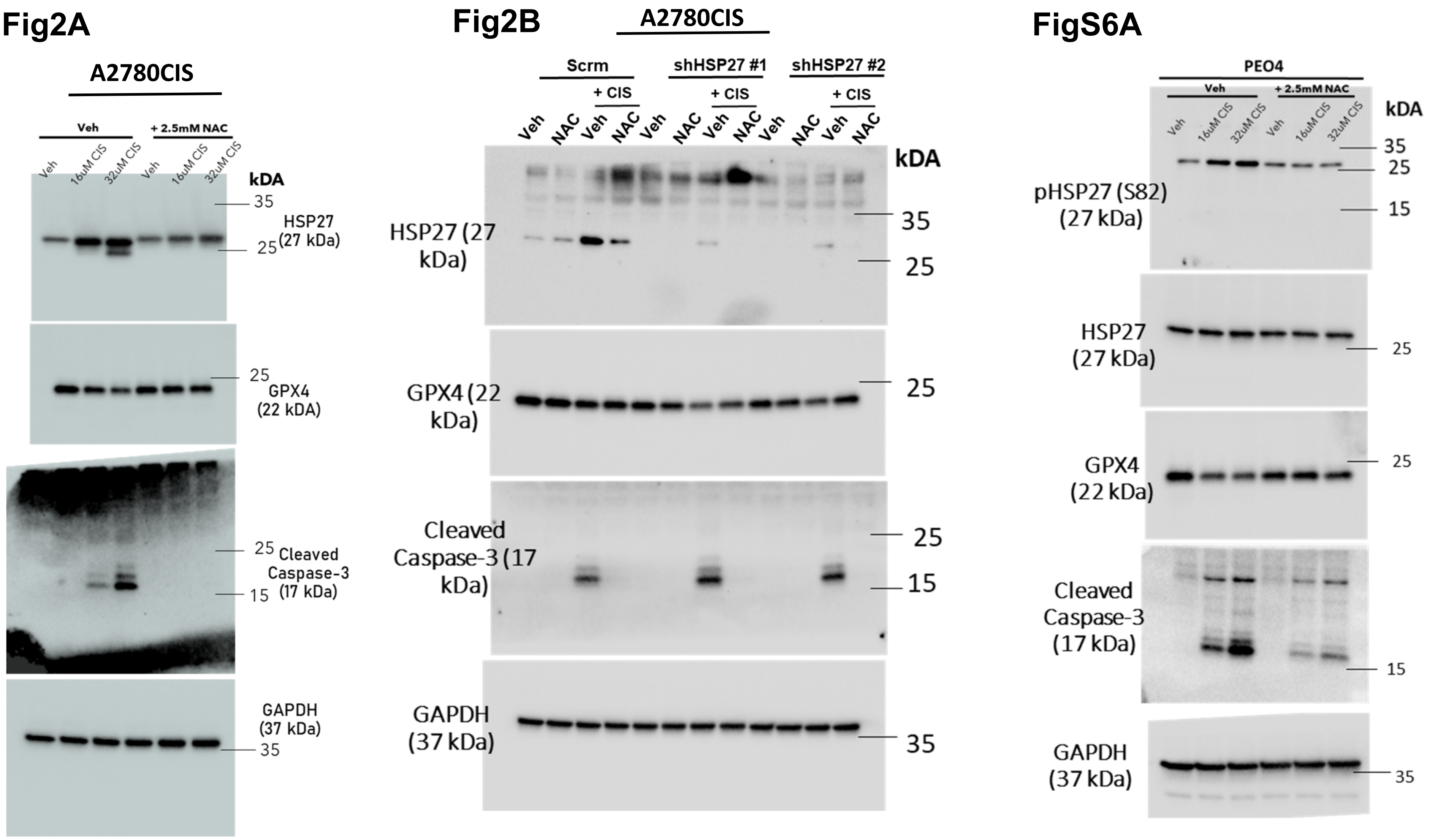


**Figure S14.** Uncropped western blot images for Fig2A, Fig2B, FigS6A

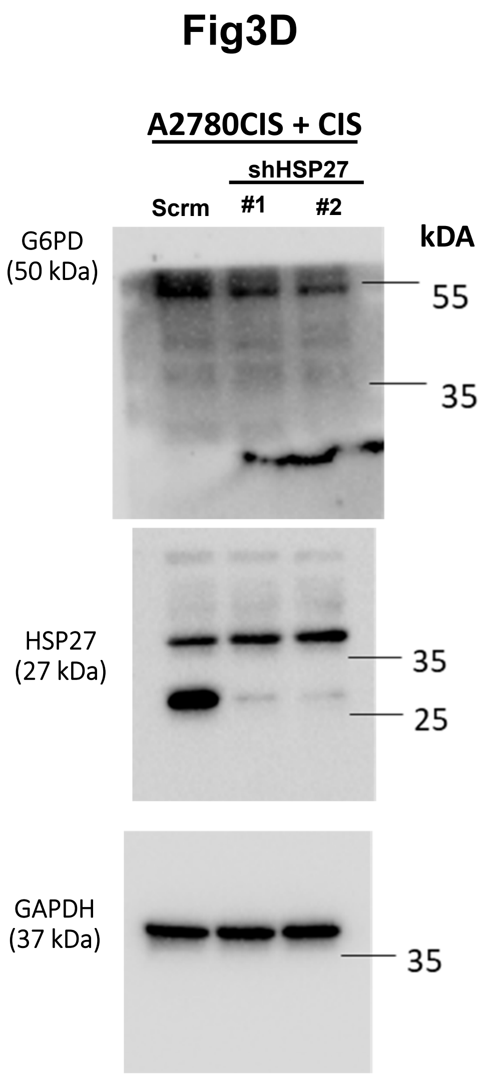


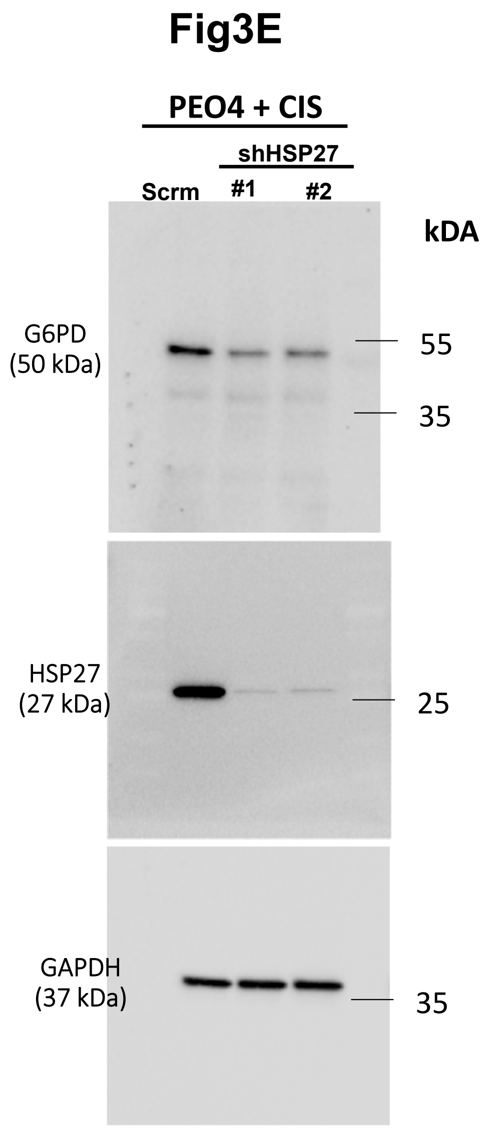


**Figure S15.** Uncropped western blot images for Fig3D, Fig3E

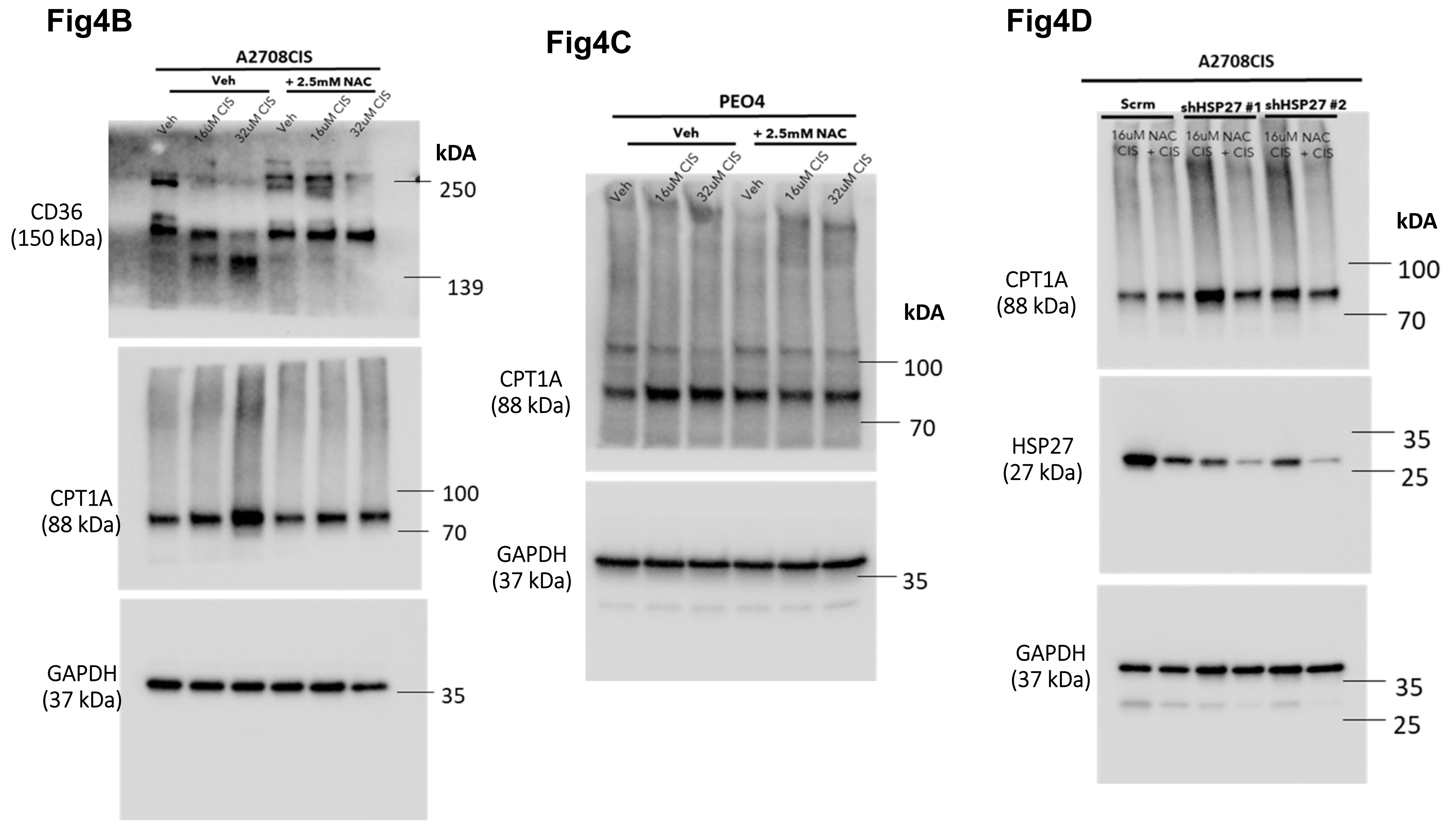


**Figure S16.** Uncropped western blot images for Fig4B, Fig4C, Fig4D

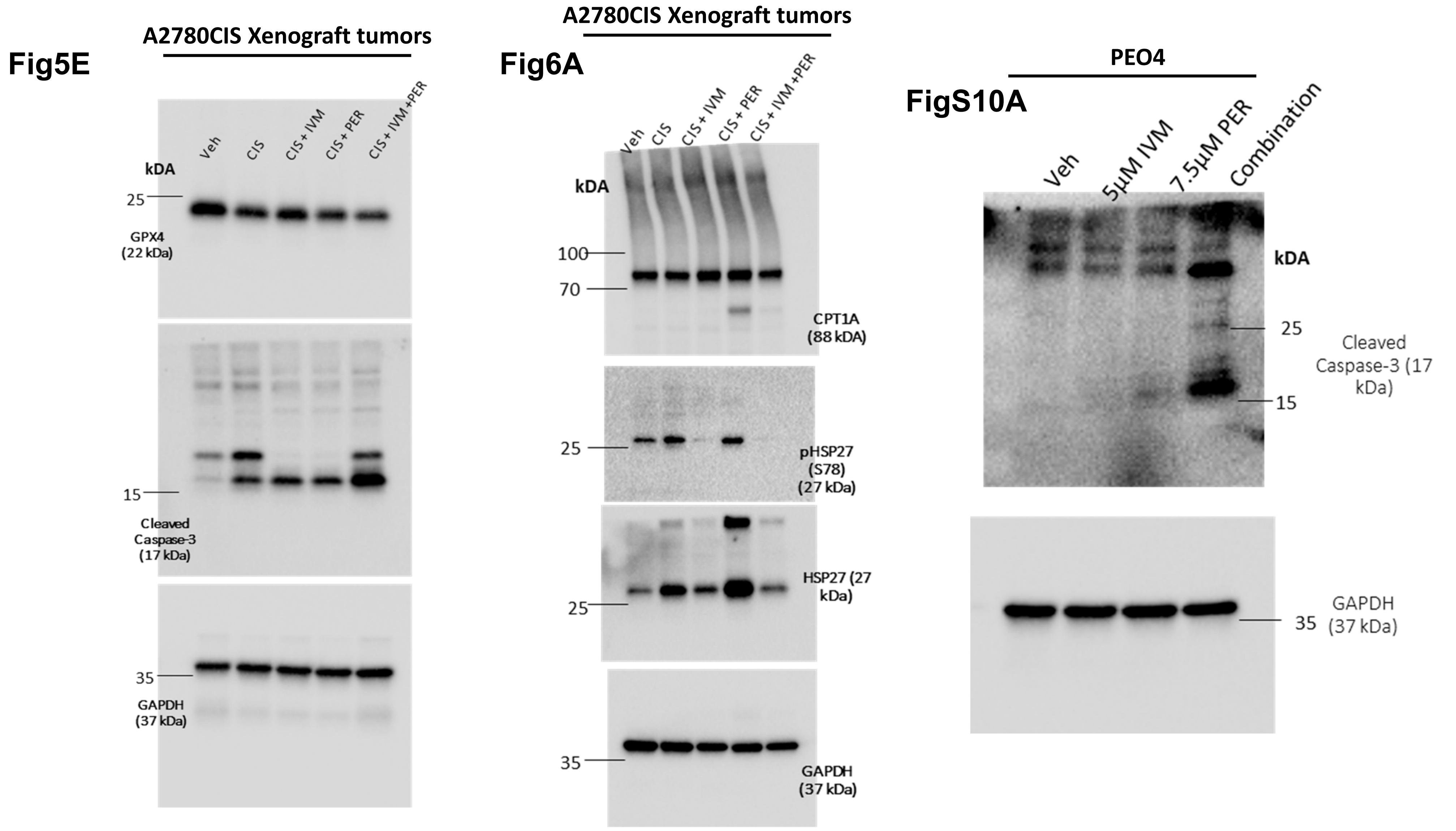


**Figure S17.** Uncropped western blot images for Fig5E, Fig6A, FigS10A
