# Supplementary material for: Targeting Heat Shock Protein 27 and Fatty Acid Oxidation Augments Cisplatin Treatment in Cisplatin-Resistant Ovarian Cancer Cell Lines
Source: Int J Mol Sci. 2023 Aug 10;24(16):12638. doi: 10.3390/ijms241612638 (PMC10454186; doi:10.3390/ijms241612638)
Supplement: Supplementary file 1 [file ijms-24-12638-s001.zip › ijms-2501187-SI/Supplementary table 1 reagent list.pdf]

Table S2. List of antibodies, kit, and reagents

Antibody, kit or reagent	Product #	Company
Phospho-HSP27 (Ser78) antibody	#2405	Cell Signaling Technology
Phospho-HSP27 (Ser82) antibody	#9709	Cell Signaling Technology
HSP27 antibody (WB)	#2402	Cell Signaling Technology
HSP27 antibody (IF)	#ab32501	Abcam
Cleaved caspase-3 antibody	#9664P	Cell Signaling Technology
GPX4 antibody	#52455	Cell Signaling Technology
G6PD antibody	#12263	Cell Signaling Technology
CPT1A antibody	#ab128568	Abcam
CD36 antibody	#ab133625	Abcam
GAPDH antibody	#10R-G109a	Fitzgerald
Goat-anti rabbit secondary antibody, FITC-conjugated	#31569	Invitrogen
Alexa Fluor 594 goat anti-rabbit Secondary antibody, IgG	#A11037	Invitrogen
DAPI	#62248	Thermo Scientific
Cisplatin	#P4394	Sigma Aldrich
Ivermectin	#I8898-25mg	Sigma Aldrich
N-Acetyl-L-cysteine	#A9165	Sigma Aldrich
Perhexiline	#SML0120-10MG	Sigma Aldrich
MitoSOX	#M36008	Invitrogen

CellROX	#C10444	Invitrogen
ThiolTracker	#T10095	Invitrogen
Image-iT Lipid peroxidation dye	#C10445-A	Invitrogen
